# Repeated Exposure to Lidocaine Induces Alzheimer's‐Like Cognitive Impairment and Neuropathology in Aged Mice Through BDNF‐Regulated Autophagy

**DOI:** 10.1111/jcmm.70970

**Published:** 2025-11-29

**Authors:** Yongxin Huang, Yanhua Guo, Honghong Zhang, Jianhui Deng, Zhibing Huang, Andi Chen, Xuyang Wu, Daoyi Lin, Peng Ye, Xiaohui Chen, Xiaochun Zheng

**Affiliations:** ^1^ Shengli Clinical Medical College of Fujian Medical University, Department of Anesthesiology, Fujian Provincial Hospital Fuzhou University Affiliated Provincial Hospital Fuzhou China; ^2^ Fujian Emergency Medical Center, Fujian Provincial Key Laboratory of Critical Care Medicine Fujian Provincial Co‐Constructed Laboratory of “Belt and Road” Fuzhou China

**Keywords:** astrocyte, autophagy, BDNF, cognitive decline, lidocaine

## Abstract

Lidocaine is widely used for perioperative pain management, but repeated exposure may cause neurotoxicity, including neurological deficits. This study investigates mechanisms underlying cognitive decline induced by repeated lidocaine exposure. Eighteen‐month‐old mice received repeated clinically relevant lidocaine infusions over 3 days. Cognitive function was assessed by Morris water maze, Y‐maze and open field tests. Hippocampal pathology was examined via TEM, Nissl staining, immunofluorescence for astrocyte polarisation and Aβ deposition, and western blot for tau, BDNF, TrkB, mTOR and autophagy proteins. The TrkB agonist 7,8‐DHF was used to modulate BDNF/TrkB/mTOR signalling. Repeated lidocaine exposure impaired cognition and induced Alzheimer's‐like hippocampal pathology, as evidenced by increased accumulation of Aβ and tau toxic proteins, along with neuronal death. It reduced BDNF expression, inhibited TrkB phosphorylation, and activated mTOR signalling, leading to autophagy inhibition and pathological protein accumulation. Lidocaine shifted astrocytes towards the neurotoxic A1 phenotype, decreasing neuroprotective A2 astrocytes and BDNF synthesis. TrkB agonist treatment restored signalling, enhanced autophagy and improved cognitive deficits and pathology. Repeated lidocaine exposure promotes A1 astrocyte increase and A2 decrease, inhibiting autophagy via the BDNF/TrkB/mTOR pathway, resulting in toxic protein deposition and Alzheimer's‐like cognitive impairment.

## 1 Introduction

Over the past several decades, the demographic transition worldwide has seen a dramatic rise in life expectancy, which is anticipated to continue escalating in the future [1]. This longevity revolution has corresponded with a rapid progression of population ageing, contributing to a surge in the number of elderly patients undergoing repeat surgeries rather than single procedures [2]. Both the natural ageing process and surgical interventions contribute to an augmented inflammatory response [3], which constitutes a significant risk factor for the development of Alzheimer's disease (AD) [4]. Moreover, older patients are increasingly prone to preexisting AD or its development [5]. Preclinical studies have shown that anaesthetics can exacerbate cognitive decline, including AD. It is essential to have greater awareness of cognitive impairment associated with anaesthesia and surgical procedures when treating older adult patients. Careful consideration should be given to the choice of anaesthetics, dosage and administration frequency [6].

Lidocaine is an amide local anaesthetic that finds widespread application in clinical practice. Its primary use is for skin and mucosal anaesthesia, peripheral nerve blockade and spinal anaesthesia [7]. Preoperatively, an intravenous bolus of lidocaine was administered, complemented by a continuous infusion throughout the surgical procedure, aimed at alleviating postoperative pain [8], reducing airway complications, [9] accelerating patient recovery and shortening hospitalisation time. Despite this, there is still uncertainty about the effects of lidocaine on cognitive function [10]. Some studies have shown that injection of lidocaine does not affect the incidence of postoperative cognitive dysfunction (POCD) in cardiac surgery patients and that high doses of lidocaine can even serve as an independent risk factor for POCD [11]. However, lidocaine can also act to reduce the inflammatory response in certain patients [12]. These studies suggest that the amount, frequency and duration of lidocaine use have various effects on cognitive function. However, given that older adult patients require lidocaine to alleviate pain, improve prognosis and are more vulnerable to adverse effects due to their age [13], as well as emergency traumas and conditions like urinary calculi [14], which increase the likelihood of adverse reactions. Furthermore, in previous studies, we confirmed that repeated exposures to lidocaine can lead to significant cognitive impairment in elderly mice without affecting their vital signs [15]. It becomes imperative to investigate the impacts and mechanisms by which repeated exposure to lidocaine influences cognitive function and the potential development of AD‐like manifestations in this population.

Macroautophagy, also known as autophagy, is a major cellular pathway that aggregates unwanted protein materials within autophagosomes and subsequently degrades them in lysosomes [16]. Dysregulated autophagy has been implicated in the pathogenesis of a wide range of neurodegenerative disorders [17]. Patients with AD show enhanced activation of the mechanistic target of the mammalian target of rapamycin (mTOR) signalling pathway, a critical regulator of autophagy that leads to reduced levels of autophagy [18, 19]. Autophagy plays a critical role in preventing the development of various neurodegenerative diseases, including AD [20]. Furthermore, local anaesthetics, such as lidocaine, can induce cytotoxicity by altering autophagy levels [21, 22].

Brain‐derived neurotrophic factor (BDNF) functions as a crucial member of the neurotrophic factor family, which is essential for the differentiation of neural stem cells (NSCs), as well as for the development, maturation and maintenance of neurons. BDNF and its receptor tropomyosin receptor kinase B (TrkB), are key modulators of dendritic growth in the central nervous system. After BDNF binding, TrkB is internalised and continues to signal within endosomes. Axonal signalling enhances the activity of mTOR kinase, which is crucial for the translation of most proteins and the expression of the plasticity‐related gene Arc. Therefore, the BDNF/TrkB/mTOR pathway is involved in the coordination of gene transcription and translation, which involves the products of genes associated with learning and memory regulation [23]. A recent study indicates that activated astrocytes play a crucial role in the supply of BDNF following the injury to the brain. Activated astrocytes have two subtypes: A1 astrocytes produce and release neurotoxic factors, mediate the classical complement cascade, and amplify neuroinflammation, whereas A2 astrocytes up‐regulate the expression of various neurotrophic factor proteins, including BDNF, by increasing their expression and release, thus exerting neuroprotective effects [24]. As a result, therapeutic strategies that promote A2 astrocytes and inhibit A1 astrocytes after brain injury can activate the BDNF/TrkB/mTOR signalling pathway, thus exerting neuroprotective effects.

This study aims to explore the specific mechanisms through which repeated exposures to lidocaine result in cognitive impairment and Alzheimer‐like symptoms. It will also examine the impact of astrocyte polarisation on autophagy and the deposition of toxic proteins.

## 2 Materials and Methods

### 2.1 Animals and Ethics Statement

The Animal Protection and Utilisation Committee of Fujian Medical University approved this study (FJMU IACUC 2021‐0431). We followed the Guidelines for the Care and Use of Laboratory Animals in managing the experimental animals. Animals were housed in a facility with controlled temperature (24°C–25°C) and under a 12:12 light–dark cycle. They were provided with ad libitum food and water during the experimental period. This study used a total of 248 animals.

### 2.2 Calculation of Animal Sample Size

In animal experiments, determining the sample size is crucial as it directly affects the reproducibility and reliability of the study results. A sample size that is too small might result in false negative or false positive conclusions, while a sample size that is too large could lead to unnecessary animal use and ethical issues, which go against the 3R principles: Replacement, Reduction and Refinement.

In this study, it is challenging to accurately estimate the standard deviation and effect size involved in behavioural. In such situations, the resource equation provides an effective way to estimate sample size [25]. This method calculates the total sample size by setting the range for the degrees of freedom of error through analysis of variance, making it suitable for exploratory animal research. The calculation formula is: E = *N* − K = K(*n* − 1); where *N* is the total sample size, *n* is the sample size of each group and K is the number of treatment groups. If the degrees of freedom of error (E) satisfy 10 ≤ E ≤ 20, then the sample size set for the study is reasonable and scientific. This formula can be simplified to: *n* = E/K + 1, allowing the minimum and maximum number of animals per group to be calculated: *n* Min = 10/K + 1, *n* Max = 20/K + 1.

According to the above plan, in the first part of this study, the number of treatment groups is 2, with a minimum and maximum number of animals per group ranging from 6 to 11. Based on actual conditions, the study ultimately included eight animals per group for analysis, resulting in E = 2 × (8−1) = 14, satisfying 10 ≤ E ≤ 20. Therefore, the sample size set in this study is reasonable and scientific.

### 2.3 Experimental Protocol for Aged Mice

Firstly, we divided the 18‐month‐old C57BL/6J mice into two groups: (1) Control group (Con) and (2) Repeated lidocaine group (Rep lido). The Y‐maze and open field experiments used the same batch of mice and were conducted on the second day following a 3‐day continuous injection of lidocaine. Hippocampal samples were collected after the experiments were completed. The Morris water maze experiment used a different batch of mice. However, due to time constraints, no sample collection and verification were performed after the experiments.

Next, in the rescue experiment, we used TrkB selective activator 7,8‐Dihydroxyflavone (7,8‐DHF) (HY‐W013372, MCE, United States). We divided the elderly mice into three groups: (1) Vehicle group (Veh group), (2) Repeated lidocaine group, (3) Rep lido model mice treated with 7,8‐DHF (Rep lido + 7,8‐DHF group). The experimental methods are consistent with those in the first part.

### 2.4 Drug Injection

The study utilised an elderly mouse model that received repeat intravenous injections of lidocaine. Each mouse was first weighed and recorded prior to being placed in a mouse restrainer with unobstructed breathing, and its tail passing through the hole at the end of the apparatus. Subsequently, the tail was immersed in 40°C–45°C warm water for 60 s, promoting dilation of the tail vein, then dried with cotton and wiped with 75% alcohol to achieve full vein dilation. Using a 26 G venous catheter pre‐loaded with lidocaine or reference solution, the catheter was slowly inserted parallel to the tail's direction into the lateral tail vein of the C57 mouse until a clear sense of empty space was felt and blood return was observed. A small amount of the solution was then slowly injected from the syringe to verify that the catheter was placed in the tail vein. If there was no apparent resistance or swelling, the catheter was secured with adhesive tape and connected to the syringe attached to a micro‐infusion pump. Clinically, the initial dose of lidocaine is 1.5 mg·kg^−1^, with a maintenance dose of 1.0 mg·kg^−1^·h^−1^, and the conversion ratio between humans and mice according to the latest guidelines is 1:12.30. The dosage for the mice was determined by considering inter‐species differences and following the latest guidelines for conversion between human and animal doses [26]; the repeat lidocaine group underwent continuous infusion of 1% lidocaine at a rate of 18.45 mg·kg^−1^, followed by a continuous infusion of 12.30 mg·kg^−1^·h^−1^ for 2 h, repeated over 3 days. The control group was continuously infused with saline at the same rate and duration.

Rep lido+7,8‐DHF group was administered an intraperitoneal injection of 7,8‐DHF (5 mg/kg) [27] for two consecutive days starting from the second day after lidocaine infusion.

### 2.5 Experimental Protocol for HT22 Hippocampal Neurons and U251 Astrocytes

The U251 astrocyte cell lines and HT22 hippocampal neuron cell lines were procured from the Cell Resource Center of the Shanghai Institutes for Biological Sciences (Shanghai, China). Both cell lines were cultured in DMEM medium (Invitrogen, Carlsbad, CA, USA) supplemented with 10% FBS (Gibco Industries Inc., Grand Island, NY, USA), 100 μg/mL streptomycin, 100 U/mL penicillin and 2 mM L‐glutamine (Invitrogen, Carlsbad, CA, USA) at 37°C under 5% CO_2_ humidified conditions.

Given their analogous traits to astrocytes, U251 cells are frequently employed as surrogates for astrocytes or in conjunction with primary astrocytes in research settings [28], while the HT22 cell line consists of immortalised neuronal cells [29]. Researchers commonly use these two cell lines as a substitute for primary cells in numerous studies about neurodegenerative diseases, including the establishment of cell co‐culture [30].

### 2.6 HT22/U251 Co‐Culture and Drug Exposure

The HT22/U251 co‐culture system was established using a transwell setup as previously described. U251 cells were seeded onto transwell inserts coated with a polylysine/poly (methyl methacrylate) membrane with a pore size of 0.4 μm at a density of 2 × 10^5^ cells per insert. The inserts were placed into 24‐well plates (Corning, NY, USA) above a monolayer of HT22 cells. Both U251 and HT22 cells were exposed simultaneously to 2.7 mM lidocaine for 2 h daily over three consecutive days. After 72 h of co‐culture, the HT22 cells in the lower chamber were harvested for analysis.

For comparison, pure HT22 cultures without co‐culture were also exposed to 2.7 mM lidocaine using the same exposure regimen. This allowed assessment of the differential impact of lidocaine on neurons alone versus neurons co‐cultured with astrocytes.

### 2.7 Nissl Staining

We evaluated the neuronal loss and morphology of hippocampal CA1 neurons using Nissl staining. Following the millimetre wave experiment, we euthanised all mice and promptly collected their brain tissue. Afterwards, we conducted paraffin embedding and sectioning according to the aforementioned method. Then, we stained the dewaxed and dehydrated sections with cresyl violet for 5–10 min, which was followed by two 10 s rinses in distilled water. Lastly, after two consecutive 5 min dehydration steps in ethanol and xylene, we mounted them on glass slides. We employed an optical microscope to observe and analyse the quantity and morphology of the neurons.

### 2.8 Western Blot

The hippocampal tissues were exposed to lidocaine for 3 days. Afterwards, they were placed on ice and homogenised using a lysis buffer containing proteinase and phosphatase enzymes. Subsequently, the protein samples were divided into equal amounts (40 g per well) and transferred onto a polyvinylidene fluoride membrane (EMD Millipore) using sodium dodecyl sulphate‐polyacrylamide gel electrophoresis. Subsequently, the membrane was blocked for 1.5 h using a solution of 5% nonfat milk diluted in Tris‐buffered saline with Trion X. Subsequently, the membrane was incubated with the primary antibody overnight at 4°C. The following day, the membrane was incubated with the secondary antibody at room temperature for 2 h. Anti‐BDNF (ab108319), Anti‐TrkB (phosphor Y705) (ab229908), Anti‐TrkB (ab187041), Anti‐mTOR (phosphor S2448) (ab109268), Anti‐mTOR (ab134903), Anti‐beta Actin (ab8227) were from Abcam. Anti‐tau (13‐6400), Anti‐tau (phosphoSer202, Thr205) (MN1020), Anti‐p62527800 (MA5‐27800), Anti‐Beclin‐1 (MA5‐15825) and Anti‐LC3B (PA5‐32254) were from Cell Signalling Technology. The protein bands were visualised and captured using the GE Amersham Imager 600 along with an enhanced chemiluminescence substrate kit.

### 2.9 Immunofluorescence Staining

For the specific experimental purposes, we utilised hippocampal slices obtained from elderly mice and cultured astrocytes for conducting immunofluorescence staining. After subjecting elderly mice to three consecutive days of lidocaine exposure, their brains were collected and fixed overnight in 4°C paraformaldehyde. Subsequently, coronal slices of brain tissue were embedded in paraffin with a thickness ranging from 3.0 to 3.5 μm. The chosen slices underwent dewaxing and hydration processes, followed by treatment with a 0.1 M borate buffer (pH 8.5) to neutralise the culture medium. Subsequently, the slices were exposed to 2 N HCL at 37°C for 30 min to induce DNA denaturation. The slices were then blocked with 3% foetal bovine serum for 3 min, subjected to three washes with phosphate‐buffered saline, and finally incubated overnight with the primary antibody. Anti‐GFAP (ab279289), Anti‐C3 (ab182890) and Anti‐S100A10 (ab76472) were from Abcam. Anti‐beta Amyloid (1‐42) (44‐344) was from Cell Signalling Technology. The following day, adequate secondary antibodies were incubated at room temperature for a duration of 2 h, subsequently followed by DAPI counterstaining. Fluorescence images were acquired using a fluorescence microscope (IX71, Olympus, Tokyo, Japan) and subsequently analysed for their fluorescence intensity using ImageJ.

U251 cells were cultured in medium supplemented with 2.7 mM lidocaine for a continuous period of 2 h per day over the course of 3 days. Subsequently, the cultured cells were seeded onto 8‐well chamber slides (Millipore, Carrigtwohill, Ireland) at a density of 2 × 10^4^ cells per well. Following that, they were fixed with 4% paraformaldehyde and subsequently blocked with 3% foetal bovine serum. The subsequent protocol steps matched those used for the hippocampal tissue slices. Lastly, proficient laboratory technicians performed manual cell counting of positive cells using a confocal laser scanning microscope (FV10i, Olympus, Tokyo, Japan) in conjunction with the cell counting function of ImageJ.

### 2.10 Transmission Electron Microscope (TEM)

After intravenous injection of pentobarbital sodium (5 mg/100 g) anaesthesia, the perfusion with 2.5% glutaraldehyde and fixation with 4% paraformaldehyde were performed. Then euthanasia was performed a second time for atlantoaxial dislocation. Approximately 1 mm^3^ hippocampal tissue was sliced and incubated at 4°C for 2 h. After fixation with 1% osmium tetroxide, the slices were treated with uranyl acetate solution. Then, the slices were dehydrated, embedded in epoxy resin and treated with lead citrate. Images were observed under a TEM (HT7700; Hitachi, Japan).

### 2.11 Quantitative Real‐Time PCR (RT‐qPCR) Analysis

Total RNA was extracted from hippocampal tissue using TRIzol reagent (Invitrogen, Waltham, MA, USA) according to the manufacturer's instructions. Complementary DNA (cDNA) was synthesised using the Superscript III RT‐PCR kit (Invitrogen, Waltham, MA, USA). Quantitative PCR was performed using TB Green Premix Ex Taq II (Takara Bio Inc., Shiga, Japan) with 1 μg of total RNA.

The primer sequences used for RT‐qPCR were as follows:

**Table:** 

Gene	Forward primer (5′–3′)	Reverse primer (5′–3′)
CD68	CGAGCATCATTCTTTCACCAGCT	ATGAGAGGCAGCAAGATGGACC
IL‐6	ACTCACCTCTTCAGAACGAATTG	CCATCTTTGGAAGGTTCAGGTTG
TNF‐α	ATGAGCACTGAAAGCATGATC	AAAGTGCAGCAGGCAGAAGA
Lcn2	CCACCTCAGACCTGATCCCA	CCCCTGGAATTGGTTGTCCTG
Gpc6	TCTCATTGCAGGGGAACACT	TCTCACAGGCAAAGCACAAA
Sparcl1	GTGAAGGCAACATGAGGGTGCA	GTTGGAGGACAAGTCACTGGATC
GADPH	GGCACAGTCAAGGCTGAGAATG	ATGGTGGTGAAGACGCCAGTA

PCR cycling conditions included an initial denaturation at 95°C for 30 s, followed by 40 cycles of 95°C for 5 s and 60°C for 34 s. Relative mRNA expression levels were normalised to GAPDH and calculated using the 2^−ΔΔCt^ method.

### 2.12 Assessment of Cell Viability

Cell viability was assessed using the Cell Counting Kit‐8 (CCK‐8, Dojindo, Kumamoto, Japan). U251 cells were seeded at a density of 2 × 10^3^ cells/well in a 96‐well microplate. The cells were then treated with increasing concentrations of Lidocaine (1–5 mM) and exposed to Lidocaine‐containing media for 2 h per day over a period of 3 days. Following the treatments, 10 μL of CCK‐8 solution was added to each well and incubated for 2–4 h. Subsequently, the absorbance at 450 nm was then measured (Biotek Instruments Inc., Vermont, USA). The data were expressed as a percentage of the control values.

### 2.13 Morris Water Maze Test

We used the Morris water maze (MWM) to assess hippocampus‐dependent spatial learning and memory, testing the hypothesis that 3 days of repeated lidocaine exposure impairs spatial navigation. The MWM test comprised a 5 days place navigation test followed by a probe test on the 6 days with a total duration of 6 days. Cameras positioned above the water pool recorded the movements of the mice. The pool was partitioned into four quadrants, one of which housed a 10 cm diameter platform. Before the formal test, the mice were given 90 s to freely swim in the pool. During the place navigation trial, the mice were positioned in each of the four quadrants of the pool with their heads oriented towards the wall. Each mouse had 60 s to freely explore the pool. Mice that independently found and climbed onto the platform were given a 15 s stay before being lifted out of the pool, dried and placed back into their cages. The time required to locate and climb onto the platform was designated as the latency period, serving as an assessment of the mice's spatial learning ability. Mice that did not find the platform within 60 s were guided to it and permitted a 15 s stay, with their latency period recorded as 60 s. Training sessions were conducted in each quadrant with a minimum 1 h interval, totaling 4 training sessions per day for five consecutive days, to measure the average escape latency and evaluate spatial learning and memory abilities. The escape platform was removed from the pool on the 6 days, during the spatial exploration experiment. The mice were softly introduced into the water, facing the wall from the central region of the fourth quadrant, which was farthest from the platform, and given 60 s for free swimming. For each mouse, the swimming distance, latency period and number of crossings through the initial platform location were documented, while the path of the mice was captured and analysed utilising a computer video‐tracking system.

### 2.14 Open Field Test

The open field experiment was conducted to detect changes in motor behaviours and similar anxiety‐like responses that might affect cognitive outcomes; we hypothesised that lidocaine would not increase anxiety levels. The assessment of locomotor activity in mice was conducted using the open field test (OFT), carried out within a rectangular arena measuring 60 cm by 40 cm, enclosed by walls 20 cm in height. Prior to the commencement of the test, mice were gently introduced to the testing environment for a period of 1 h to facilitate environmental adaptation. Each mouse was positioned at the centre of the open field arena and granted 5 min of unsolicited exploration. The cumulative distance traversed by the mice within the confinements of the box was automatically documented and subjected to detailed analysis through the utilisation of a video‐tracking software, specifically, Smart Version 2.5.20. To uphold the integrity of experimental conditions and mitigate cross‐contamination, the experimental arena underwent sanitisation with 75% ethanol subsequent to the examination of each individual mouse.

### 2.15 Y Maze

The Y‐maze spontaneous alternation test indexes short‐term memory and cognitive flexibility, under the hypothesis that repeated lidocaine exposure reduces alternation performance, reflecting hippocampus‐related memory impairment. All mouse groups were subjected to the Y‐maze test to assess working memory impairment. The maze consisted of three arms, each measuring 30 × 6 × 15 cm. During the test, each mouse was placed in one arm and allowed to freely explore the maze for 5 min. The alternation rate was calculated using the following formula: (actual alternation/maximum alternation) × 100%. The actual alternation value was defined as the number of times the mouse entered all three arms in succession, while the maximum alternation value was calculated as the total number of arm entries minus two. In the Y‐maze test, a lower alternation rate indicates impaired working memory.

### 2.16 Histology and Image Analysis/Quantification

#### 2.16.1 Sections Per Animal and Sampling Scheme

Sections per animal and FOVs per section: For each ROI, we analysed 15 coronal sections per animal (thickness 20 μm) sampled at a fixed interval. Within each section, we acquired fields of view from the hippocampal CA1 region at 35× magnification using a grid‐based scheme.

#### 2.16.2 Imaging Consistency

All images were captured on the same microscope (FV10i, Olympus, Tokyo, Japan) with identical acquisition parameters across groups (laser power, detector gain, exposure, pinhole) determined during pilot calibration to avoid saturation. Daily calibration with a standard slide was performed, and sample imaging order was randomised and interleaved across groups to minimise drift.

#### 2.16.3 Positivity Criteria

For Aβ‐42 and other markers, positivity was defined a priori using negative controls (no‐primary/isotype) to set fixed thresholds (intensity plus size) that were applied uniformly across all images. For fluorescence, thresholds were determined by [mean + SD] and held constant within batches.

#### 2.16.4 Blinding

Image acquisition, ROI selection and quantification were performed under blinded conditions. Slides were coded by an independent team member, and group labels were revealed only after final data export. We recognise that our previous description did not make this sufficiently clear, and we apologise for the omission.

#### 2.16.5 Reporting and Analysis

We now report exact *n* values at the animal, section and FOV levels in the Methods and figure legends. Quantification was performed in Fiji with scripted pipelines, and animal‐level means were used as the unit of inference. Where appropriate, we used ANOVA and controlled for multiple comparisons.

### 2.17 Statistical Analysis

Data analysis was performed using Graphpad Prism 9.0 or SPSS 25.0, and the results were presented as mean ± standard deviation. Statistical analyses in this study included the Student's *t*‐test, one‐way analysis of variance (ANOVA) and repeated measures two‐way analysis of variance.

## 3 Results

### 3.1 Repeated Lidocaine Exposure Induced Cognitive Impairment, Neurotoxic Proteins Deposition and Neural Damage in Aged Mice

**FIGURE 1:**
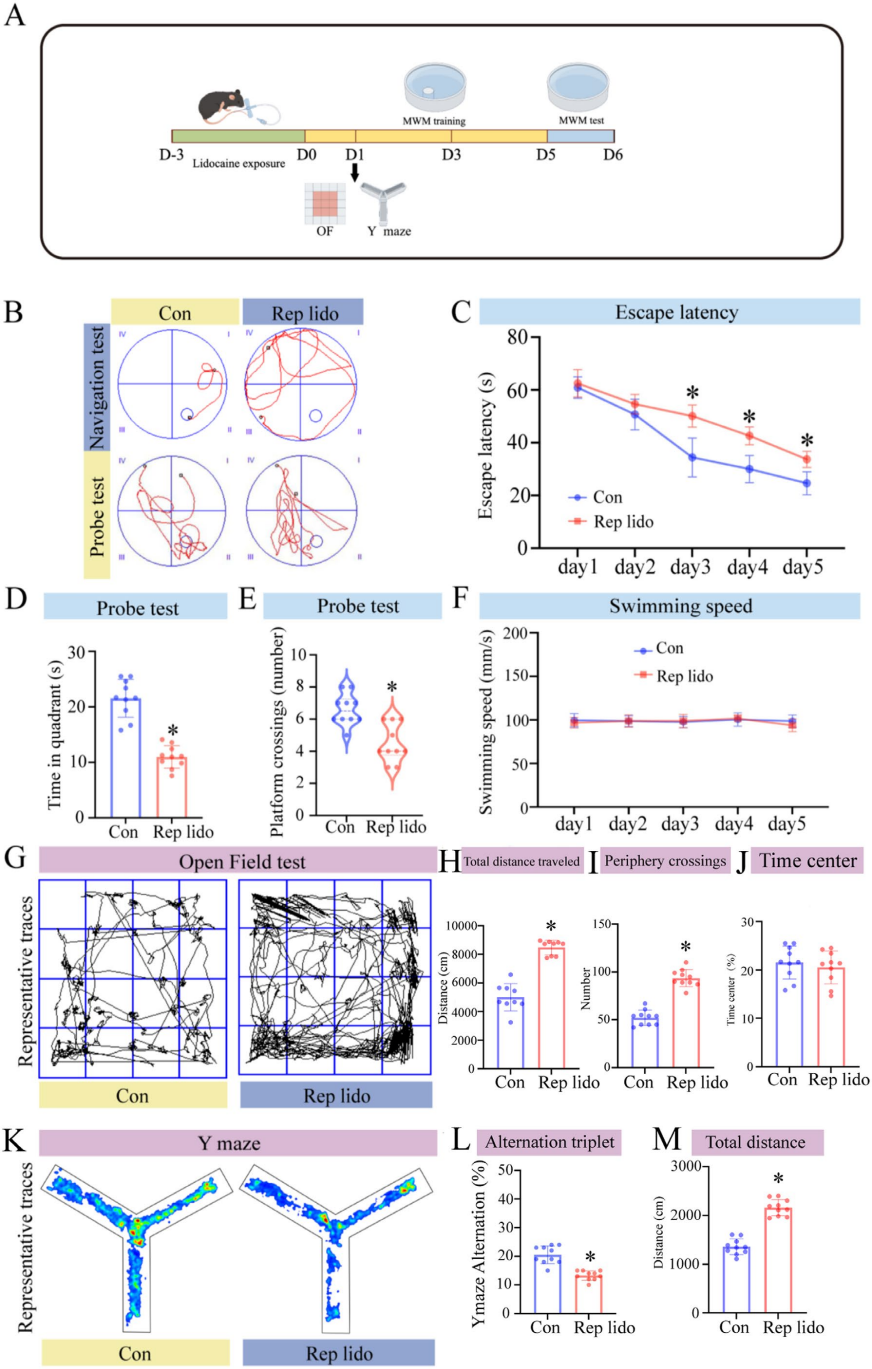
Lidocaine‐induced cognitive impairment in aged mice. (A) Behavioural study flowchart. (B) Representative swimming trajectories of the mice during the place navigation experiment on day 5 and the probe trial on day 6. (C) The average escape latency to reach the concealed platform was measured throughout the place navigation test on days 1–5. (D) Time spent in the target quadrant during the probe test. (E) Number of platform crossings during the probe test. (F) Swimming speeds of the mice in two groups during the probe test. (G) Representative traces in the OFT. (H) Total distance travelled by mice during the testing period. (I) Number of periphery crossings. (J) Time spent in the center zone during the OFT. (K) Representative traces in the Y maze. (L) Alternation triplet in Y maze. (M) Total distance travelled by mice during the testing period. Data were expressed as the mean ± SD (*n* = 10 per group); *p < 0.05 vs. the Con group.

We evaluated the effects of repeated lidocaine exposure (Rep lido group) on spatial learning and memory in aged mice using the MWM test, Y‐maze and OFT. The experimental design is shown in Figure 1A. Figure 1B presents a representative diagram of the MWM test. The MWM was used to assess hippocampus‐dependent spatial learning and memory, testing the hypothesis that repeated lidocaine exposure impairs spatial navigation. As shown in Figure 1C, during the 5 days navigation test, the escape latency of all groups decreased rapidly (*p* < 0.05), and there was no significant interaction between training days or groups (*p* > 0.05), indicating that spatial learning and memory abilities improved with time. On days 1 and 2, there were no significant differences in escape latency between the Con and Rep lido groups. However, on days 3–5 of the experiment, the escape latency of the Rep lido group was significantly longer than that of the Con group. This suggests that repeated lidocaine treatments caused cognitive impairment. In the probe test on day 6, the number of platform crossings of the Rep lido group of mice was significantly reduced (*p* < 0.05; Figure 1D), and the time spent in the target quadrant was significantly less than that of the Con group (*p* < 0.05; Figure 1E), indicating learning and memory impairment. Furthermore, there were no significant differences in swimming speed between the groups (*p* > 0.05; Figure 1F), indicating that the observed differences in the results of the MWM test were due to changes in cognitive function. To delve further into the cognitive impact, OFT were administered on days 1, 3 and 5 post‐lidocaine injection and the Y‐maze experiment was performed on day 3. The representation of OFT is illustrated in Figure 1G. We conducted the OFT to assess potential changes in locomotor activity and anxiety‐like behaviour that could confound cognitive outcomes. Compared to the Con group, the Lido group exhibited significantly increased overall activity, as evidenced by greater total distance travelled and a higher number of locomotor bouts (*p* < 0.05; Figure 1H,I). However, no significant differences were observed in the time spent in the central area, indicating unaltered anxiety levels (Figure 1J). Intriguingly, certain cognitive impairment models, such as APP/PS1 mice with early‐stage Alzheimer's disease, also show hyperactivity characterised by increased total movement in the open field, despite maintained short‐term memory and anxiety‐related behaviour [31]. This suggests that hyperactivity can coexist with cognitive deficits. Our findings of increased activity in lidocaine‐treated mice align with this phenomenon. Both groups demonstrated habituation during the session, with the Lido group maintaining a higher activity level throughout. The Y‐maze spontaneous alternation test is a well established behavioural assay for assessing working memory and cognitive flexibility. The schematic of the Y‐maze is presented in Figure 1K. Our results showed a significant reduction in the spontaneous alternation rate in the Lido group (*p* < 0.05; Figure 1L), along with a marked increase in total locomotor distance (*p* < 0.05; Figure 1M). In line with the OFT findings, where locomotor activity was elevated without alterations in centre‐related parameters, there was no evidence of increased anxiety levels. Therefore, the decreased spontaneous alternation likely reflects impaired cognitive flexibility rather than anxiety or diminished motor ability. Notably, the difference in alternation between groups remained statistically significant, supporting the interpretation that lidocaine exposure impairs hippocampus‐dependent short‐term memory.

**FIGURE 2:**
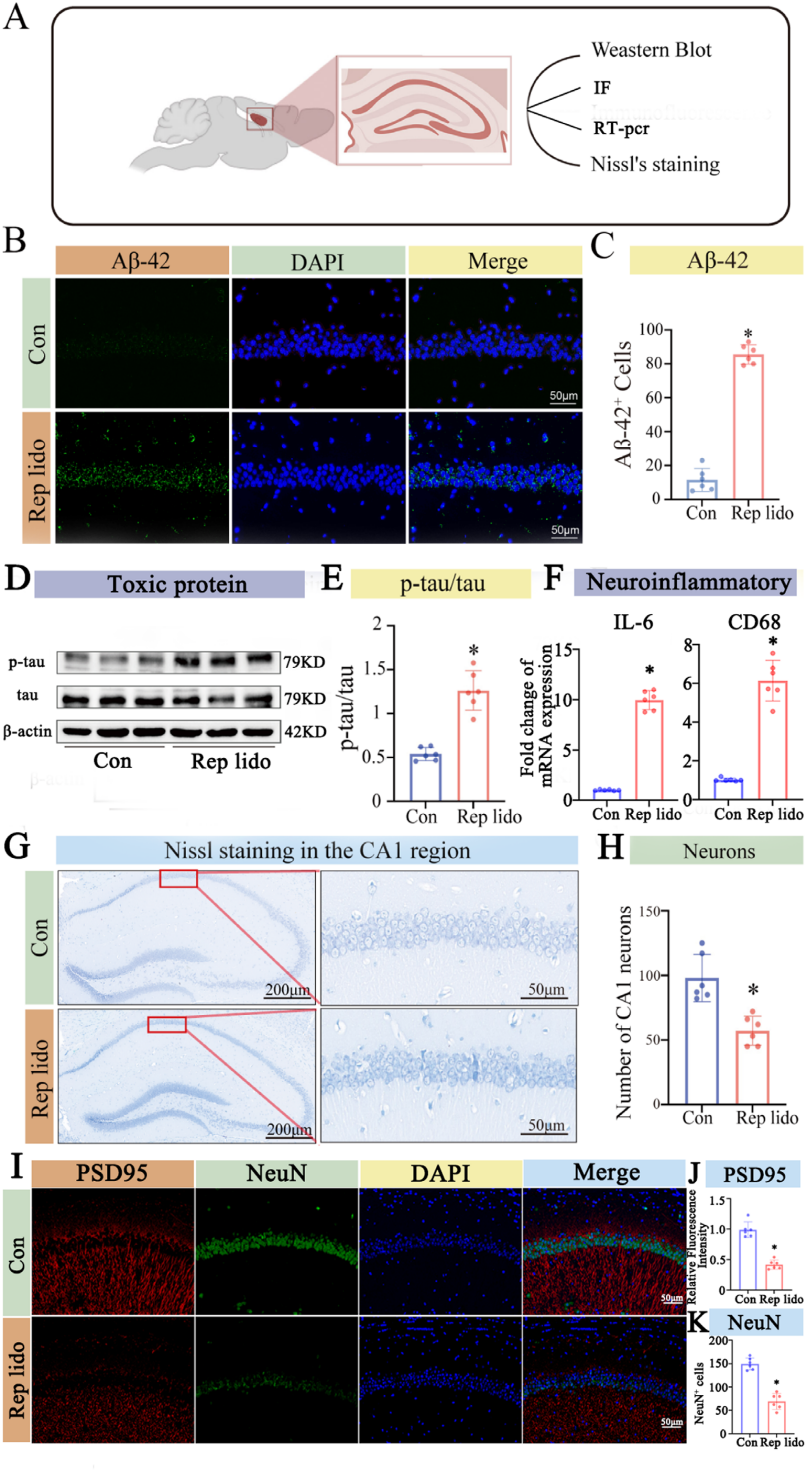
Lidocaine induced Alzheimer's‐like pathologic symptoms in old mice. (A) Schematic diagram. (B) Immunofluorescence staining was performed to examine the amount of Aβ‐42 deposition in hippocampus. (C) Quantification of the number of CA1 Aβ‐42 positive cells in the hippocampus. (D) Western blot analysis was used to measure tau phosphorylation levels in the hippocampus of both the Con group and the Rep lido group. (E) The statistical densitometric analysis showing tau phosphorylation levels. (F) Quantitative PCR analysis of CD68 and IL‐6 mRNA expression levels. (G) Nissl staining was used to examine neuronal count in the CA1 region of the hippocampus in both the Con group and the Rep lido group. (H) Quantitative analysis of the CA1 of the hippocampal region is shown in (G). (I) Immunofluorescence staining was performed to detect the fluorescence intensity of PSD95 and the number of Neun‐positive cells in the hippocampal CA1 region. (J) Quantitative analysis of the average fluorescence intensity of PSD95 in the CA1 region of the hippocampus was conducted. (K) Quantification of the number of CA1 Neun positive cells in the hippocampus. Data were expressed as the mean ± SD (*n* = 6 per group); *p < 0.05 vs. the Con group.

The experimental protocol depicted in Figure 2A was employed to investigate the effects of repeated lidocaine injections on Alzheimer's disease‐related pathology in aged mice. First, we assessed the deposition of hippocampal amyloids by immunofluorescence. Our findings indicate that the Rep lido group caused a significantly higher Aβ‐42 deposition in the CA1 region of the hippocampus than the Con group (*p* < 0.05; Figure 2B,C). Additionally, Western blot analysis detected a significant elevation in tau protein phosphorylation in the Rep lido group (*p* < 0.05; Figure 2D,E). Meanwhile, PCR analysis indicated that the levels of neuroinflammation‐related molecules IL‐6 and CD68 were also significantly increased (*p* < 0.05; Figure 2F). Notably, Nissl staining revealed fewer neurons in the Rep lido group (*p* < 0.05; Figure 2G,H). To further validate the condition of neurons, we conducted additional analyses using immunostaining for PSD95 and neuronal nuclear protein (NeuN) to quantify synapses and neuron counts. The results indicated that both the neuron count and the average fluorescence intensity of synapses were significantly reduced compared to the control group (*p* < 0.05; Figure 2I–K). The findings align with previous research, demonstrating that an increased Aβ load, tau protein hyperphosphorylation and neuroinflammation are linked to neuronal loss in neurodegenerative diseases [32, 33]. Accordingly, the present results align with our hypothesis that repeated lidocaine exposure induces AD‐like pathology and cognitive impairment in aged mice.

### 3.2 Repeated Lidocaine Exposure Inhibits the Autophagy and BDNF/TrkB/mTOR Signalling Pathway in Aged Mice

**FIGURE 3:**
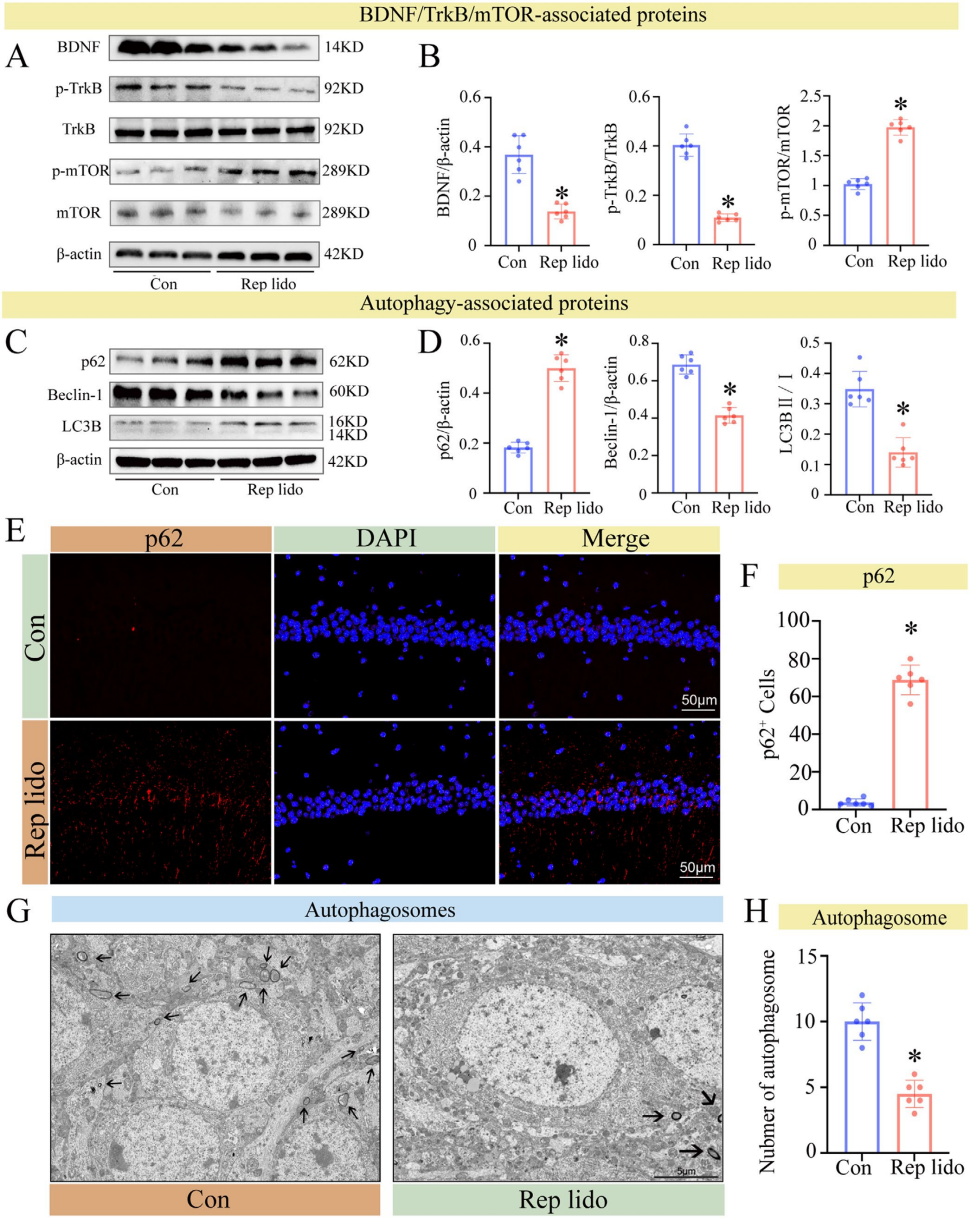
Lidocaine inhibited the BDNF/TrkB/mTOR signalling pathway and reduced autophagy. (A) BDNF, TrkB phosphorylation and mTOR phosphorylation levels in the hippocampus were detected by Western blot. (B) The statistical densitometric analysis showing the levels of BDNF, TrkB phosphorylation and mTOR phosphorylation. (C) The levels of p62, Beclin‐1 and LC3B levels in the hippocampus were detected by Western blot. (D) The statistical densitometric analysis showed the levels of p62, Beclin‐1 and LC3B. (E) Immunofluorescence staining was performed to examine the number of p62‐positive cells in hippocampal CA1 area. (F) Quantification of the number of CA1 p62 positive cells in the hippocampus. (G) Transmission electron microscopy of hippocampal tissue sections. (H) Number of hippocampal autophagosomes in each group in the TEM. Data were expressed as the mean ± SD (*n* = 6 per group); *p < 0.05 vs. the Con group.

The mTOR signalling pathway is closely associated with autophagy [34]. We first used Western blot to quantify protein expression levels of BDNF, p‐TrkB, TrkB, p‐mTOR and mTOR in hippocampal tissue. Our findings indicate that, compared to the control group, BDNF and TrkB phosphorylation levels decreased significantly in the Rep lido group, while the levels of mTOR phosphorylation increased significantly (*p* < 0.05; Figure 3A,B).

The deposition of toxic proteins is closely related to the level of autophagy [35]. To assess autophagy levels, Western blot, IF and TEM were used. For WB analysis, we selected p62, Beclin‐1 and LC3B as key markers: p62 acts as a selective autophagy substrate that accumulates when autophagy is impaired; Beclin‐1 is essential for autophagosome initiation; and the conversion of LC3B from LC3B‐I to LC3B‐II indicates autophagosome maturation. The Western blot results revealed that the expression of Beclin‐1 and the LC3B‐II/I ratio was significantly reduced in the group receiving repeated lidocaine injections, while p62 protein levels increased, indicating reduced autophagy (*p* < 0.05; Figure 3C,D). We used IF to detect autophagic flux mediated by p62 protein [36] (Figure 3E), and consistent results were obtained with Western blot. In the Rep lido group, p62 protein increased significantly (*p* < 0.05; Figure 3F). TEM results showed that the number of autophagosomes in the group that received repeat injections of lidocaine was significantly reduced (*p* < 0.05; Figure 3G,H). Therefore, the administration of repeat injections of lidocaine reduced autophagy levels in aged mice. This observation explicates the emergence of Alzheimer's disease‐related pathologies in aged mice, wherein a reduction in autophagic function curtails their efficacy in eliminating toxic protein accumulations [37].

### 3.3 Repeated Lidocaine Exposure Prompted Astrocyte Polarisation Towards A1 and Reduced A2 Astrocyte Derived‐BDNF In Vivo and In Vitro

**FIGURE 4:**
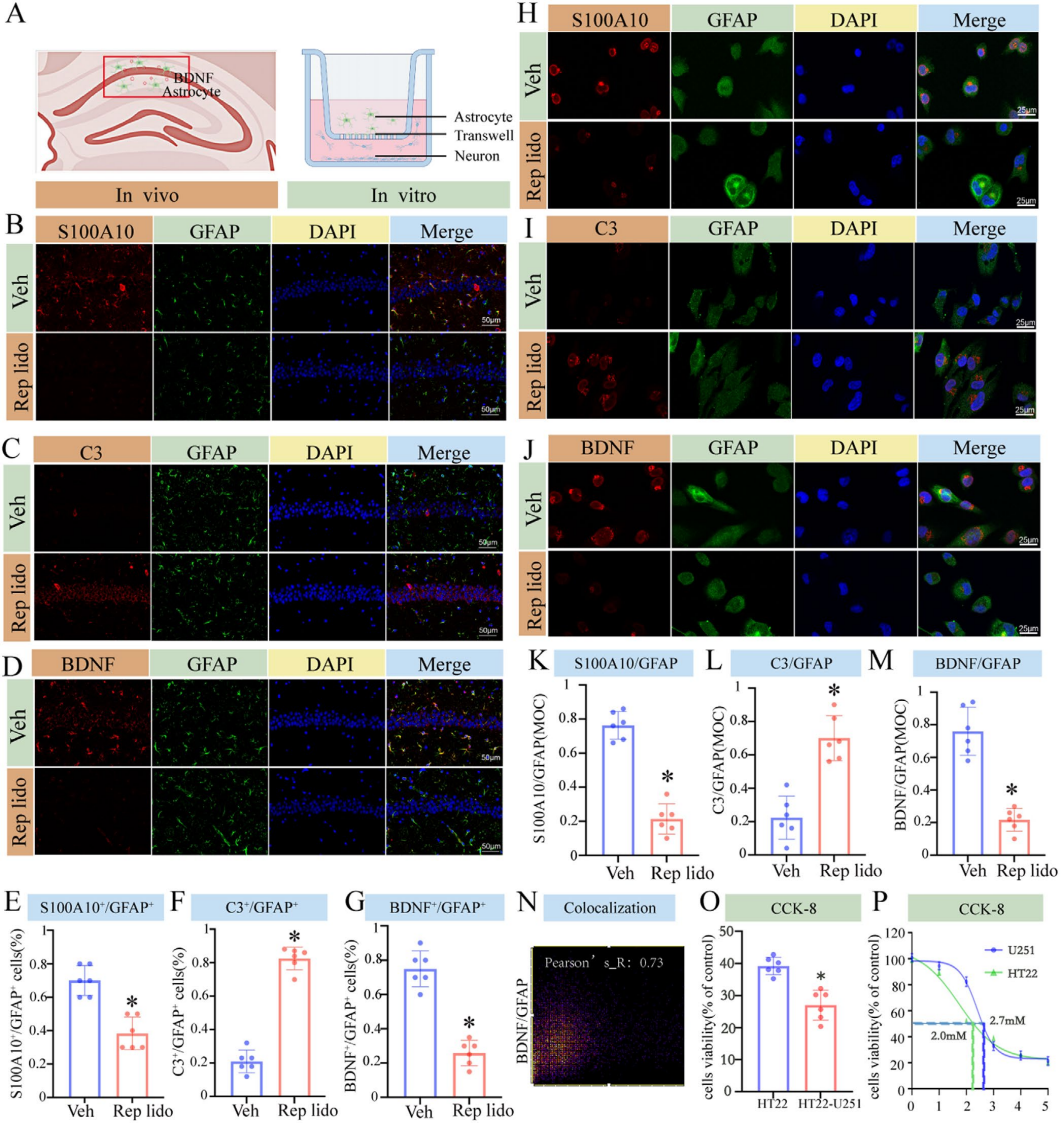
Lidocaine modulated BDNF expression by altering astrocyte polarisation in both cellular models and in aged mice. (A) Schematic diagram. (B–D) Dual immunofluorescence staining was performed to detect the quantity of S100A10, C3, and BDNF (Red) and GFAP (Green) in hippocampal slices. (E–G) Quantification of the number of S100A10, C3 and BDNF positive cells in the hippocampus CA1 region. (H–J) Dual‐immunofluorescence staining was performed to detect the amount of S100A10, C3, and BDNF (red), and GFAP (green) in the U251 cell line. (K‐M) The intensity of fluorescence of S100A10, C3 and BDNF protein was quantified using ImageJ. (N) Colocalization between BDNF and GFAP was determined by Pearson correlation coefficient. (O) The viability of HT22 cells was evaluated both individually and within a co‐culture system with U251 cells following prolonged exposure to lidocaine. (P) U251 and HT22 cells were treated with Lido (0, 1.0, 2.0, 3.0, 4.0 and 5.0 mM) for 2 h daily, continuously for 3 days. Cell viability was determined by the CCK‐8 assay. Data were expressed as the mean ± SD (*n* = 6 per group); *p < 0.05 vs. the Con group.

As shown in Figure 4A, we performed dual validation both in vivo and in vitro. First, we employed an IF assay to assess BDNF expression and identify activated astrocyte subtypes in the hippocampus of aged mice subjected to repeated lidocaine injections, as well as in cultured U251 astrocytes. Treatment with Rep lido resulted in a substantial rise in the population of A1 astrocytes concomitant with a decline in A2 astrocytes within the hippocampus of aged mice. Furthermore, it elicited a notable suppression of BDNF expression in the hippocampal region (Figure 4B–G). To further validate changes in astrocyte subtypes, we performed RT‐qPCR analysis on established molecular markers related to A1 and A2 astrocyte identification (TNF‐α and Lcn2 for A1; Gpc6 and Sparcl1 for A2). These results, presented in Figure S1A–D, provide additional evidence supporting astrocyte polarisation shifts in response to repeated lidocaine exposure. Importantly, immunofluorescence staining with GFAP and C3 antibodies across the entire brain revealed upregulation of GFAP and C3 expression in multiple brain regions, including the hippocampus. However, due to the hippocampus's central role in learning, memory and spatial navigation, as well as its complex neural networks that are indispensable for cognitive function, it is widely recognised as a critical target region for research on cognitive impairment. Therefore, this study focuses on the hippocampus to elucidate the mechanisms underlying lidocaine‐induced cognitive dysfunction.

Consistent with the findings from in vivo experiments, the Rep lido group resulted in a decrease in the population of A2 astrocytes and the expression of BDNF, while simultaneously increasing the number of A1 astrocytes. Notably, the expression of BDNF was almost completely co‐localised with that of S100A10 (Figure 4H–M), yielding a Pearson correlation coefficient of 0.73, which indicates significant co‐localisation with GFAP (Figure 4N). Furthermore, we compared the viability of HT22 cells exposed to lidocaine alone with that of HT22 cells co‐cultured with U251 cells, revealing a notably lower viability of HT22 cells under the co‐culture condition. This observation suggests that astrocytes exacerbate neuronal death upon exposure to lidocaine, highlighting a potential role for these cells in mediating lidocaine‐induced neurotoxicity (Figure 4O). In our in vitro experiments, we employed an exposure model analogous to the in vivo experiments. Cells were continuously exposed to a medium containing lidocaine for 3 days, after which the IC50 values for HT22 and U251 were determined (Figure 4P). These findings support the notion that repeat injections of lidocaine can have a profound impact on neuronal cells, presumably by altering the astrocyte subtypes present and influencing the expression of BDNF.

### 3.4 Activation of the BDNF/TrkB/mTOR Signalling Pathway Promoted Autophagy in Aged Mice Following Lidocaine Treatment

**FIGURE 5:**
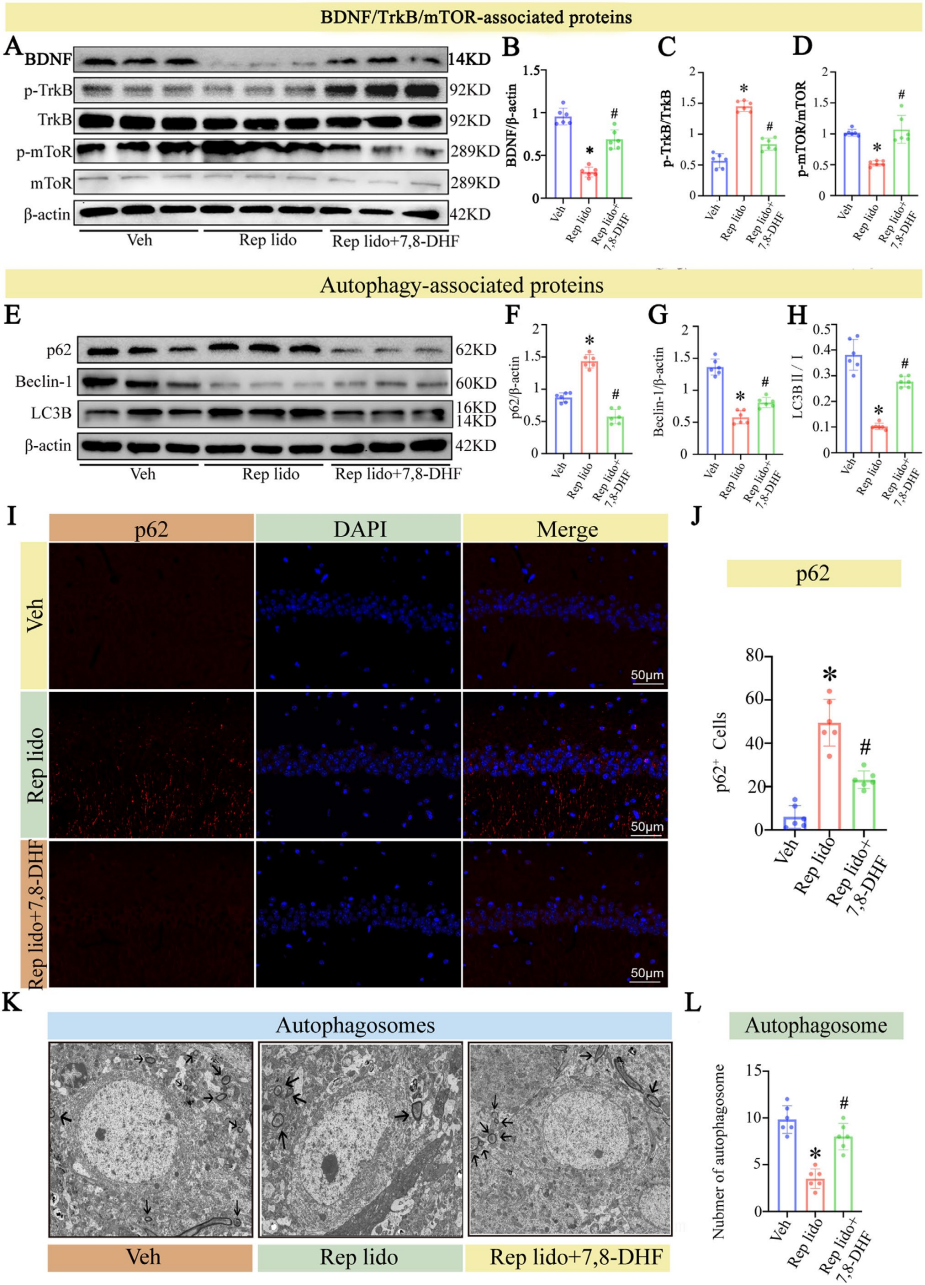
Activated the BDNF/TrkB/mTOR pathway alleviated lidocaine‐induced autophagy suppression in aged mice. (A) BDNF, TrkB and mTOR phosphorylation levels in the hippocampus were detected by Western blot. (B–D) The statistical densitometric analysis showing the levels of BDNF, TrkB phosphorylation and mTOR phosphorylation. (E) The levels of p62, Beclin‐1 and LC3B in the hippocampus were detected by Western blot. (F–H) The statistical densitometric analysis showing the levels of p62, Beclin‐1 and LC3B. (I) Immunofluorescence staining was performed to examine the number of p62‐positive cells in the CA1. (J) Quantification of the number of CA1 p62‐positive cells in the hippocampus. (K) TEM of hippocampal tissue sections. (L) The number of hippocampal autophagosomes in each group visualised by TEM. Data were expressed as the mean ± SD (*n* = 6 per group); *p < 0.05 vs. the Con group; ^#^ *p* < 0.05 vs. the Rep lido group.

To verify whether the BDNF/TrkB/mTOR signalling pathway is a direct molecular mechanism underlying Rep lido‐mediated reduction of autophagy, we used the TrkB selective agonist, 7,8‐DHF, to activate the BDNF signalling pathway, as shown in Figure 5A. In the Rep lido +7,8‐DHF group, the phosphorylation levels of BDNF and TrkB were significantly higher compared to the Rep lido group. Conversely, the phosphorylation level of mTOR was significantly lower in the Rep lido +7,8‐DHF group (Figure 5B–D). These findings suggest that our activator and injection method are both precise and effective, and that the TrkB activator can substantially enhance BDNF expression. Subsequently, we evaluated autophagy levels in the three groups by Western blot (Figure 5E–H). The level of autophagy in the Rep lido +7,8‐DHF group was significantly higher than that in the Rep lido group. Simultaneously, we used immunofluorescence to determine autophagy flux (Figure 5I,J) and TEM to examine the number of autophagosomes in the three groups (Figure 5K,L). These findings suggest that activation of the BDNF/TrkB/mTOR signalling pathway can alleviate the inhibitory effects of Rep lido on autophagy in ageing mice.

### 3.5 Activation of the BDNF/TrkB/mTOR Signalling Pathway Reduced the Accumulation of Neurotoxic Proteins Aβ and p‐Tau and Improve Cognitive Function in Aged Mice

**FIGURE 6:**
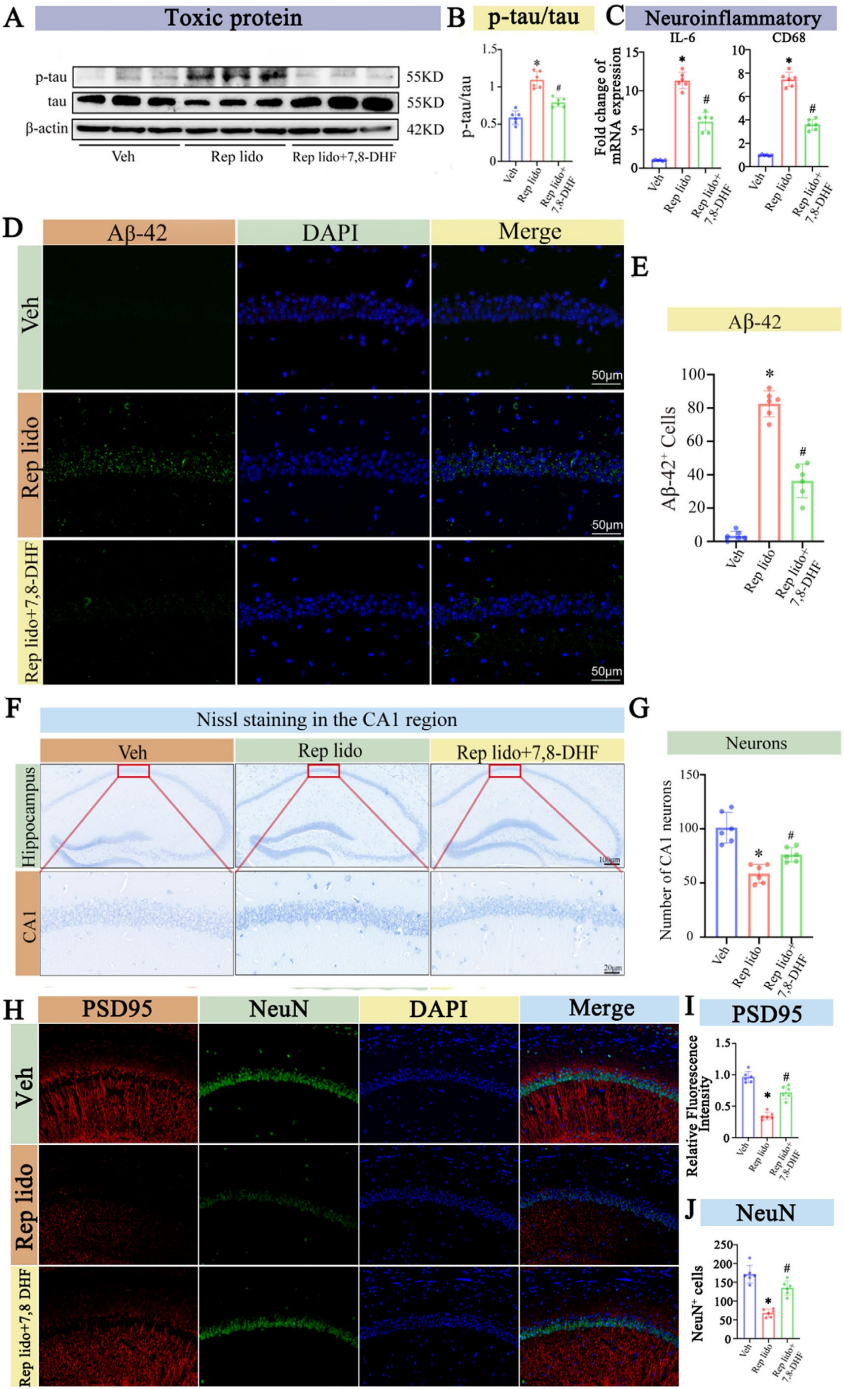
7,8‐DHF alleviated the symptoms of lidocaine‐induced Alzheimer's disease in aged mice. (A) Western blot analysis was used to measure tau phosphorylation levels in the hippocampus of both the Con group and the Rep lido group. (B) Analysis of the grey value showed that tau phosphorylation levels increased significantly in the Rep lido group compared to the Con group. (C) Quantitative PCR analysis of CD68 and IL‐6 mRNA expression levels. (D) Immunofluorescence staining was performed to examine the amount of Aβ‐42 deposition in hippocampal CA1 neurons. (E) Quantification of the number of CA1 Aβ‐42 positive cells in the hippocampus. (F) Nissl staining was used to examine the neuronal count in the CA1 region of the hippocampus in both the Con group and the Rep lido group. (G) Quantitative analysis of the CA1 of the hippocampal region is shown in (F). (H) Immunofluorescence staining was performed to detect the fluorescence intensity of PSD95 and the number of Neun‐positive cells in the hippocampal CA1 region. (I) Quantitative analysis of the average fluorescence intensity of PSD95 in the CA1 region of the hippocampus was conducted. (J) Quantification of the number of CA1 Neun positive cells in the hippocampus. Data were expressed as the mean ± SD (*n* = 6 per group); *p < 0.05 vs. the Con group; ^#^ *p* < 0.05 vs. the Rep lido group.

**FIGURE 7:**
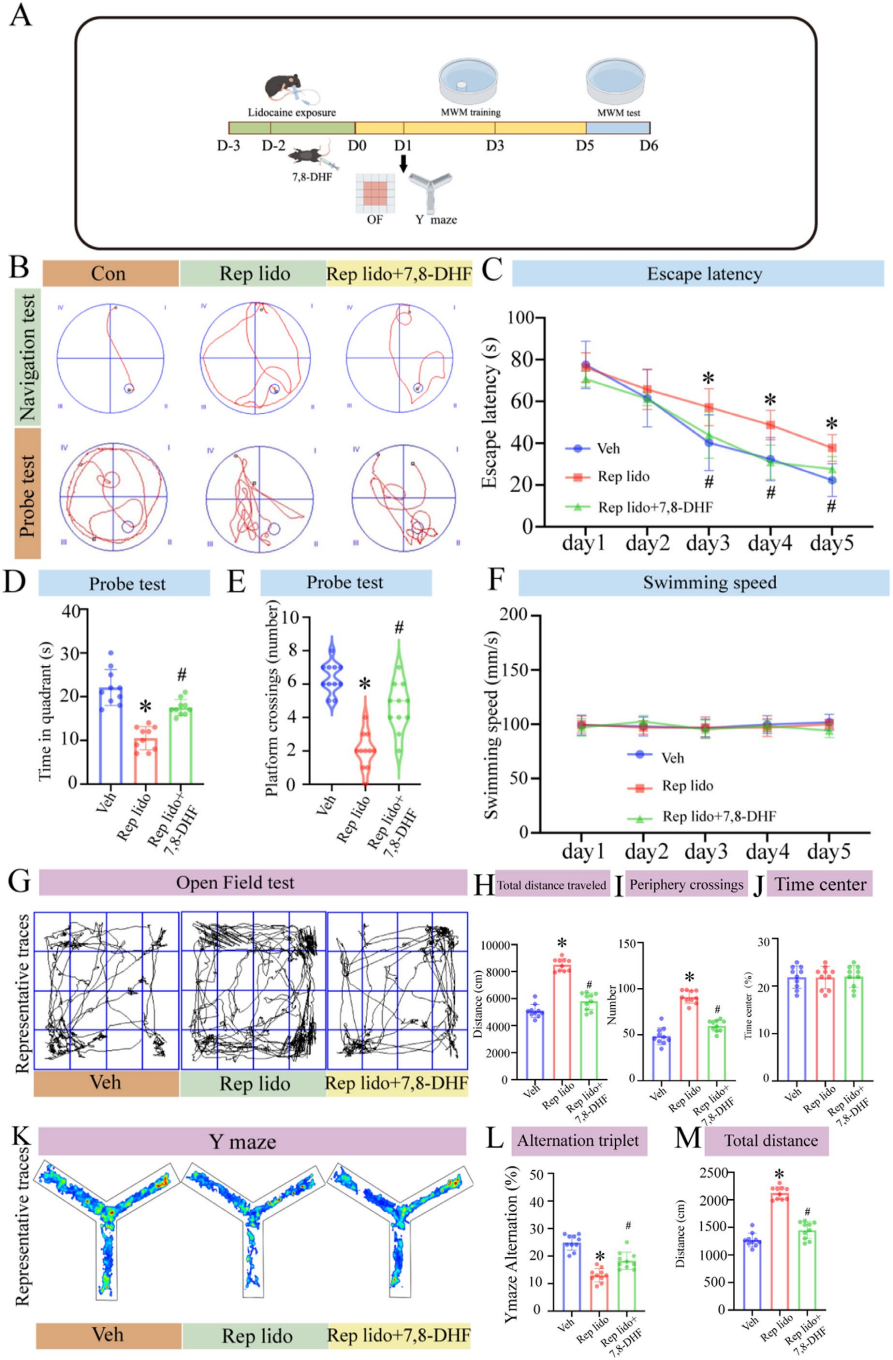
7,8‐DHF alleviated lidocaine‐induced cognitive dysfunction in aged mice. (A) Behavioural study flowchart. (B) Representative swimming trajectories of the mice during the place navigation experiment on day 5 and the probe trial on day 6. (C) The average escape latency to reach the concealed platform was measured throughout the place navigation test on days 1–5. (D) Time spent in the target quadrant during the probe test. (E) Number of platform crossings during the probe test. (F) Swimming speeds of the mice in three groups during the probe test. (G) Representative traces in the OFT (H) Total distance travelled by mice during the testing period. (I) Number of periphery crossings. (J) Time spent in the center zone during the OFT. (K) Representative traces in the Y maze. (L) Alternation triplet in Y maze. (M) Total distance travelled by mice during the testing period. Data were expressed as the mean ± SD (*n* = 10 per group); *p < 0.05 vs. the Con group; ^#^ *p* < 0.05 vs. the Rep lido group.

In this study, we found that in mice exhibiting Alzheimer's disease‐like symptoms, the phosphorylation levels of tau protein in the Rep lido +7,8‐DHF group were significantly lower than in the Rep lido group (*p* < 0.05; Figure 6A,B). Additionally, this group exhibited a significant decrease in neuroinflammation (*p* < 0.05; Figure 6C) and a reduction in Aβ‐42 deposition (*p* < 0.05; Figure 6D,E). Consistent results from Nissl staining (*p* < 0.05; Figure 6F,G) and immunostaining for NeuN and PSD95 indicated that the Rep lido +7,8‐DHF group had an increased number of neurons and enhanced synaptic growth compared to the Rep lido group (*p* < 0.05; Figure 6H–J). Furthermore, we investigated the contribution of the BDNF/TrkB/mTOR signalling pathway to Rep lido‐induced cognitive impairment in aged mice. The experimental design is shown in Figure 7A. To evaluate cognitive function, we used the MWM test, which tests the hypothesis that repeated lidocaine exposure impairs hippocampus‐dependent spatial cognition. On the first and second days of testing, the escape latency of the three groups of mice was comparable. However, on days 3–5, escape latency in the Rep lido group was longer than that in the Veh group, indicating cognitive impairment. On days 3–5, the Rep lido +7,8‐DHF group exhibited significantly shorter escape latency compared to the Rep lido group (Figure 7B). Furthermore, in the probe trial carried out on day 6, mice in the Rep lido group exhibited a shorter time spent in the target quadrant (Figure 7C) and fewer platform crossings (Figure 7D,E) relative to the Veh group, indicating the presence of memory impairment. Treatment with 7,8‐DHF alleviated both these phenomena. Our findings demonstrate that activation of the BDNF/TrkB/mTOR signalling pathway can mitigate the cognitive decline induced by Rep lido in aged mice. Importantly, the three groups of mice had similar swimming speeds (Figure 7F), indicating that the cognitive changes were not due to physical differences between the groups.

The results from the OFT further confirmed that 7,8‐DHF effectively mitigates the transient hyperactivity induced by lidocaine without exacerbating anxiety‐ or depression‐like behaviours (Figure 7G–J). Consistently, the Y‐maze test demonstrated that 7,8‐dihydroxyflavone significantly improves both cognitive deficits and transient hyperactivity caused by repeated lidocaine exposure (Figure 7K–M). These findings also support our hypothesis that lidocaine induces cognitive impairment and Alzheimer's disease‐like behaviours in aged mice by modulating the BDNF/TrkB/mTOR signalling pathway.

## 4 Discussion

Intravenous lidocaine is increasingly utilised in clinical practice to alleviate opioid‐induced pain, encompassing both perioperative and chronic pain conditions [38]. Numerous studies have demonstrated that lidocaine can influence postoperative cognitive levels, either alleviating or exacerbating cognitive impairment [39]. Notably, intravenous lidocaine has been shown to improve cognitive dysfunction in high‐risk populations, whereas in the context of video‐assisted thoracoscopic surgery (VATS), it exhibits no significant effect on postoperative cognitive function. However, a functional MRI study revealed that intravenous administration of lidocaine at clinically relevant doses not only extensively reduces functional MRI responses in acute pain regions but also inhibits memory capabilities [40]. This finding aligns with our experimental results, as our study confirmed that lidocaine suppresses memory and cognitive functions. We regret that we are currently unable to directly measure the concentration of lidocaine in the hippocampus or other brain regions. However, we initially observed that repeated administration of lidocaine led to cognitive impairment, and since the hippocampus is a pivotal brain region for cognitive function [41], it became the primary focus of our investigation. We hypothesise that the impact of intravenous lidocaine on neurocognitive function may depend on the frequency of administration and the age of patients. Studies have shown that continuous lidocaine injections can effectively reduce hospital stay duration and opioid usage [42]. Consequently, we selected elderly patients and repeat administrations to amplify and elucidate the potential neurotoxicity of lidocaine.

Behavioural experiments revealed that repeated lidocaine injections resulted in significant cognitive impairments in aged mice, accompanied by transient motor hyperactivity. This effect was primarily evidenced by increased locomotor distances observed in both open field and Y‐maze tests, while no significant changes in swimming speed were noted in the Morris water maze. The discrepancy may be attributed to differences in testing time points and durations, as the Y‐maze and OFT were conducted on the first day following lidocaine administration, whereas the water maze assessment occurred on day five. This dual phenotype suggests that lidocaine not only damages hippocampus‐dependent cognitive circuits but also modulates neural mechanisms regulating motor behaviour. Previous studies have demonstrated that lidocaine induces neuroinflammation and synaptic damage in the hippocampus, leading to cognitive deficits. Additionally, lidocaine may alter central nervous system excitability, resulting in increased locomotor activity. Such cognitive impairment accompanied by hyperactivity is not uncommon and has been reported in other neuropsychiatric animal models. For instance, mice treated with the NMDA receptor antagonist MK‐801 exhibit cognitive deficits together with marked hyperactivity [43, 44]. Similar features are observed in ketamine models and neonatal ventral hippocampal lesion models. Moreover, ADHD animal models also display coexisting cognitive dysfunction and hyperactivity [45]. These models share common features involving neurotransmitter imbalance, neuroinflammation and disrupted neural circuits, suggesting that the behavioural phenotype induced by lidocaine may result from similar pathological mechanisms. Future research should aim to further elucidate the molecular pathways through which lidocaine affects cognitive and motor neural circuits, thereby guiding safer clinical applications and minimising neurotoxic risks. Astrocytes, the most abundant cell type in the brain, play a crucial role in maintaining brain homeostasis. Astrocyte dysfunction significantly impacts neuronal survival in various neurological diseases. During the perioperative period, neuron–neuron interactions are a key feature of the ageing hippocampus [46]; however, the mechanisms and sites of action involving astrocytes and microglia in these interactions remain unclear. Repeat studies have demonstrated that anaesthetics influence neuronal activity and synaptic connectivity through glial cells, ultimately affecting cognitive function [47]. Research indicates that sevoflurane can provide neuroprotective effects by reducing astrocyte reactivity [48], while dexmedetomidine can convert neurotoxic A1 phenotype astrocytes into neuroprotective A2 phenotype astrocytes, thereby improving cognitive function. However, certain anaesthetics, such as ketamine, may exacerbate cognitive impairments [49]. In this study, we found that lidocaine induces a shift in astrocytes from the neuroprotective A2 phenotype to the neurotoxic A1 phenotype in the hippocampus of aged mice, which exacerbates cognitive injury [41]. A2 phenotype astrocytes play a vital role in supporting neuronal survival and growth, enhancing synaptic transmission and plasticity, and promoting learning and memory formation due to their ability to produce BDNF. Our research showed that repeated exposure to lidocaine reduced BDNF expression in the hippocampus of aged mice. BDNF is closely related to neuronal synaptic connectivity and plasticity [50], as neurons require interaction with astrocytes through BDNF to encode long‐term memory. Research indicates that lifestyle [51], pharmaceuticals and age [52] all influence the expression of BDNF. As individuals age, BDNF levels decline, which may contribute to the onset and progression of various neurodegenerative diseases [53]. This study employed a specific agonist of the BDNF receptor TrkB to elucidate the mechanism by which lidocaine influences BDNF, leading to Alzheimer's disease‐like pathology and cognitive decline.

Interestingly, although BDNF/TrkB signalling is typically linked with upregulating mTOR activity through the PI3K/Akt pathway, our data (Figure 3B) demonstrated a reduction in BDNF/TrkB alongside an increase in p‐mTOR following repeated lidocaine injections in aged mice. This paradoxical increase in p‐mTOR, despite decreased BDNF/TrkB, may indicate the activation of alternative or compensatory mTOR regulatory pathways independent of the canonical BDNF/TrkB axis. Under pathological or stress conditions, other factors such as inflammatory signalling [54], AMPK dysregulation [55], and cellular stress responses can induce mTOR phosphorylation, overriding normal BDNF‐dependent regulation. Similar phenomena have been observed in neuropsychiatric and neurodegenerative disorders, where abnormal mTOR activation contributes to disease progression by impairing autophagy and promoting tau phosphorylation [56]. Therefore, our findings emphasise the complexity of mTOR regulation in neurotoxic contexts, such as lidocaine exposure, and suggest that increased p‐mTOR may arise from pathways independent of BDNF mediation, warranting further mechanistic investigation. Furthermore, previous studies highlight the critical roles astrocytes play in neuroinflammation, neurotransmitter regulation, synaptic plasticity and neurotrophic support, as well as their significant impact on the development of cognitive impairment [57]. Given the complex effects of lidocaine on astrocyte polarisation, further research is imperative.

Autophagy serves a dual role in neurodegenerative diseases, with both excessive and insufficient levels potentially resulting in cognitive impairment [58]. In particular, elevated autophagy levels are considered a key mechanism in cognitive dysfunction induced by chronic sleep deprivation [59]. In contrast, in age‐related neurodegenerative diseases, a decrease in autophagy can accelerate disease progression [60]. This occurs because these diseases are characterised by increased oxidative stress, leading to the accumulation of dysfunctional organelles and proteins, which further exacerbates neuronal dysfunction or apoptosis. Normally, autophagy degrades these toxic products, thereby protecting neurons from oxidative damage and cell death. However, as the brain ages, this protective function of autophagy diminishes, making neurons more susceptible to stress stimuli [61]. Compared to normal individuals, the expression of autophagy‐related genes in AD patients is significantly reduced, leading to the pathologies of tau and Aβ. Our study indicates that lidocaine also decreases the level of autophagy in the hippocampus, resulting in the accumulation of toxic proteins and exacerbating neuronal death. In aged mice, lidocaine inhibits autophagic activity in the hippocampus. This phenomenon is evidenced by the overactivation of mTOR (a negative regulator of autophagy), reduced levels of autophagy markers such as LC3B, Beclin‐1 and p62, and decreased number of autophagosomes. In our study, astrocytes contribute to cognitive impairment through the BDNF/TrkB/mTOR signalling pathway, aligning with the findings of Yang et al. [62]. Moreover, recent research has discovered that astrocytes not only influence autophagy via BDNF but also secrete mesencephalic astrocyte‐derived neurotrophic factor (MANF), which regulates autophagy and affects ageing and other processes [63]. In neurodegenerative diseases such as AD and PD, the plasticity of autophagy in astrocytes is considered an important cellular process that affects protein degradation and cognitive function in mice [64].

Previous studies have shown that lidocaine often influences autophagy in tumour cells and reduces cancer cell proliferation by activating autophagy [65]. However, there is currently a lack of research on the effects of lidocaine on neurons and glial cells, particularly in the brain microenvironment of patients. Our research aims to fill this gap in the field. Building upon prior studies [15], this research further characterises the effects of repeated exposure to clinically safe doses of lidocaine on astrocytes; nevertheless, some limitations remain. First, our understanding of the specific molecular mechanisms by which lidocaine affects the polarisation of astrocytes is limited. We only observed changes in the number of A1/A2 astrocyte phenotypes, and exploring these mechanisms will be an interesting direction for future research. Additionally, we used agonists to detect the pathways without intervening in the upstream astrocyte polarisation and downstream autophagy, which will also be the focus of our future studies. Lastly, we used agonists instead of adenoviruses to validate the pathways, which resulted in a lack of specificity.

## 5 Conclusions

Our study demonstrates that repeat injections of lidocaine effectively increase the population of A1 astrocytes in a detrimental way and decrease the population of A2 astrocytes in a beneficial way. This results in suppression of autophagy levels through the BDNF/TrkB/mTOR signalling pathway. Consequently, it worsens the pathological symptoms associated with AD and leads to cognitive dysfunction and disease‐related symptoms in aged mice. These findings highlight the potential pharmacological mechanisms underlying the neurotoxic effects of lidocaine.

## Author Contributions

**Yongxin Huang:** writing – original draft (lead). **Yanhua Guo:** methodology (equal), writing – review and editing (equal). **Honghong Zhang:** resources (equal), software (equal). **Jianhui Deng:** software (equal), supervision (equal). **Zhibing Huang:** software (equal), visualization (equal). **Andi Chen:** investigation (equal), validation (equal). **Xuyang Wu:** formal analysis (equal), funding acquisition (equal). **Daoyi Lin:** supervision (equal), visualization (equal). **Peng Ye:** methodology (equal), validation (equal). **Xiaohui Chen:** visualization (equal), writing – review and editing (equal). **Xiaochun Zheng:** funding acquisition (lead), visualization (equal).

## Funding

This research was funded by the National Natural Science Foundation of China (Grant Nos. 82171186 and 82471278); the Joint Funds for the Innovation of Science and Technology of Fujian Province (Grant Nos. 2023Y9282 and 2023Y9315); and the Startup Fund for scientific research, Fujian Medical University (Grant numbers: 2021QH1307).

## Ethics Statement

The Animal Care and Use Committee at Fujian Medical University (Fuzhou, China) approved this research (No: FJMUIACUC2021‐0431). During the animal experiments, we followed the National Research Council's Guide for the Care and Use of Laboratory Animals. All efforts were aimed at reducing suffering and the number of experimental animals utilised.

## Consent

The authors have nothing to report.

## Conflicts of Interest

The authors declare no conflicts of interest.

## Supporting information


**Figure S1:** RT‐qPCR analysis of A1 and A2 astrocyte marker expression following repeated lidocaine exposure (A, B) Relative mRNA levels of markers specific to A1 (TNF‐α, Lcn2). (C, D) Relative mRNA levels of markers specific to A2 (Gpc6, Sparcl1). Data were expressed as the mean ± SD (*n* = 6 per group); *p < 0.05 vs. the Con group.

**Figure S2:** Effects of repeated lidocaine exposure on astrocyte activation in multiple brain regions (A–C) Representative images of GFAP and C3 staining in whole brain, cortex and hypothalamus. (D, E) Quantification of the number of GFAP and C3 positive cells in the Cortex region. (F, G) Quantification of the number of GFAP and C3 positive cells in the hypothalamus region. Data were expressed as the mean ± SD (*n* = 6 per group); *p < 0.05 vs. the Con group.

## Data Availability

The original contributions presented in the study are included in the article/[Link], [Link]; further inquiries can be directed to the corresponding author.

## References

[1] J. R. Aunan , M. M. Watson , H. R. Hagland , and K. Søreide , “Molecular and Biological Hallmarks of Ageing,” British Journal of Surgery 103, no. 2 (2016): e29–e46. 26771470 10.1002/bjs.10053

[2] D. Kelleher , K. Windle , R. Randell , et al., “A Process Evaluation of the NIDUS‐Professional Dementia Training Intervention for UK Homecare Workers,” Age and Ageing 53, no. 5 (2024): afae109. 38796316 10.1093/ageing/afae109 PMC11127770

[3] G. Eamer , A. Taheri , S. S. Chen , et al., “Comprehensive Geriatric Assessment for Older People Admitted to a Surgical Service,” Cochrane Database of Systematic Reviews 1, no. 1 (2018): Cd012485. 29385235 10.1002/14651858.CD012485.pub2 PMC6491328

[4] Y. C. Hsieh , Z. M. Augur , M. Arbery , et al., “Person‐Specific Differences in Ubiquitin‐Proteasome Mediated Proteostasis in Human Neurons,” Alzheimers Dement 20 (2024): 2952–2967. 38470006 10.1002/alz.13680 PMC11032531

[5] S. S. Arora , J. L. Gooch , and P. S. García , “Postoperative Cognitive Dysfunction, Alzheimer's Disease, and Anesthesia,” International Journal of Neuroscience 124, no. 4 (2014): 236–242. 23931049 10.3109/00207454.2013.833919

[6] K. Zeng , J. Long , Y. Li , and J. Hu , “Preventing Postoperative Cognitive Dysfunction Using Anesthetic Drugs in Elderly Patients Undergoing Noncardiac Surgery: A Systematic Review and Meta‐Analysis,” International Journal of Surgery 109, no. 1 (2023): 21–31. 36799783 10.1097/JS9.0000000000000001 PMC10389238

[7] J. Kessler , P. Marhofer , P. M. Hopkins , and M. W. Hollmann , “Peripheral Regional Anaesthesia and Outcome: Lessons Learned From the Last 10 Years,” British Journal of Anaesthesia 114, no. 5 (2015): 728–745. 25690833 10.1093/bja/aeu559

[8] X. Wu , X. Wei , L. Jiang , J. Cai , M. Ju , and X. Zheng , “Is Lidocaine Patch Beneficial for Postoperative Pain?: A Meta‐Analysis of Randomized Clinical Trials,” Clinical Journal of Pain 39, no. 9 (2023): 484–490. 37278487 10.1097/AJP.0000000000001135 PMC10399934

[9] S. S. Yang , N. N. Wang , T. Postonogova , et al., “Intravenous Lidocaine to Prevent Postoperative Airway Complications in Adults: A Systematic Review and Meta‐Analysis,” British Journal of Anaesthesia 124, no. 3 (2020): 314–323. 32000978 10.1016/j.bja.2019.11.033

[10] F. Bilotta , E. Stazi , A. Zlotnik , S. Gruenbaum , and G. Rosa , “Neuroprotective Effects of Intravenous Anesthetics: A New Critical Perspective,” Current Pharmaceutical Design 20, no. 34 (2014): 5469–5475. 24669972 10.2174/1381612820666140325110113 PMC4388052

[11] R. Y. Klinger , M. Cooter , T. Bisanar , et al., “Intravenous Lidocaine Does Not Improve Neurologic Outcomes After Cardiac Surgery: A Randomized Controlled Trial,” Anesthesiology 130, no. 6 (2019): 958–970. 30870159 10.1097/ALN.0000000000002668 PMC6520120

[12] R. Y. Klinger , M. Cooter , M. Berger , et al., “Effect of Intravenous Lidocaine on the Transcerebral Inflammatory Response During Cardiac Surgery: A Randomized‐Controlled Trial,” Canadian Journal of Anesthesia 63, no. 11 (2016): 1223–1232. 27470233 10.1007/s12630-016-0704-0 PMC7097969

[13] A. D. Ong and N. Ram , “Fragile and Enduring Positive Affect: Implications for Adaptive Aging,” Gerontology 63, no. 3 (2017): 263–269. 27974722 10.1159/000453357 PMC5391284

[14] G. A. Mashour , D. T. Woodrum , and M. S. Avidan , “Neurological Complications of Surgery and Anaesthesia,” British Journal of Anaesthesia 114, no. 2 (2015): 194–203. 25204699 10.1093/bja/aeu296

[15] X. Chen , H. Wan , Y. Huang , et al., “Repeated Lidocaine Exposure Induces Synaptic and Cognitive Impairment in Aged Mice by Activating Microglia and Neurotoxic A1 Astrocytes,” iScience 28, no. 3 (2025): 112041. 40092614 10.1016/j.isci.2025.112041 PMC11910116

[16] F. M. Menzies , A. Fleming , and D. C. Rubinsztein , “Compromised Autophagy and Neurodegenerative Diseases,” Nature Reviews. Neuroscience 16, no. 6 (2015): 345–357. 25991442 10.1038/nrn3961

[17] K. M. Chung , N. Hernández , A. A. Sproul , and W. H. Yu , “Alzheimer's Disease and the Autophagic‐Lysosomal System,” Neuroscience Letters 697 (2019): 49–58. 29758300 10.1016/j.neulet.2018.05.017

[18] H. Zhang , X. Chen , T. Zheng , et al., “Amitriptyline Protects Against Lidocaine‐Induced Neurotoxicity in SH‐SY5Y Cells via Inhibition of BDNF‐Mediated Autophagy,” Neurotoxicity Research 39, no. 2 (2021): 133–145. 33156513 10.1007/s12640-020-00299-6

[19] P. Chen , X. Chen , H. Zhang , et al., “Dexmedetomidine Regulates Autophagy via the AMPK/mTOR Pathway to Improve SH‐SY5Y‐APP Cell Damage Induced by High Glucose,” Neuromolecular Medicine 25, no. 3 (2023): 415–425. 37017880 10.1007/s12017-023-08745-2

[20] A. Caccamo , E. Ferreira , C. Branca , and S. Oddo , “Retraction Note: p62 Improves AD‐Like Pathology by Increasing Autophagy,” Molecular Psychiatry 26, no. 7 (2021): 3664. 32782379 10.1038/s41380-020-0854-x PMC7876154

[21] L. Chen , C. L. Fan , X. Zhang , S. Chen , L. Ye , and X. Zheng , “Lidocaine Induces Neurotoxicity in Spinal Cord Neurons in Goto‐Kakizaki Rats via AMPK‐Mediated Mitophagy,” Journal of Toxicological Sciences 48, no. 11 (2023): 585–595. 37914286 10.2131/jts.48.585

[22] J. L. Chen , S. T. Liu , C. C. Wu , Y. C. Chen , and S. M. Huang , “The Potency of Cytotoxic Mechanisms of Local Anesthetics in Human Chondrocyte Cells,” International Journal of Molecular Sciences 25, no. 24 (2024): 13474. 39769238 10.3390/ijms252413474 PMC11676234

[23] X. Chen , F. Gao , C. Lin , et al., “mTOR‐Mediated Autophagy in the Hippocampus Is Involved in Perioperative Neurocognitive Disorders in Diabetic Rats,” CNS Neuroscience & Therapeutics 28, no. 4 (2022): 540–553. 34784444 10.1111/cns.13762 PMC8928925

[24] J. T. Hinkle , V. L. Dawson , and T. M. Dawson , “The A1 Astrocyte Paradigm: New Avenues for Pharmacological Intervention in Neurodegeneration,” Movement Disorders 34, no. 7 (2019): 959–969. 31136698 10.1002/mds.27718 PMC6642014

[25] J. Charan and N. D. Kantharia , “How to Calculate Sample Size in Animal Studies?,” Journal of Pharmacology and Pharmacotherapeutics 4, no. 4 (2013): 303–306. 24250214 10.4103/0976-500X.119726 PMC3826013

[26] C.‐H. Koo , J. Baik , H. J. Shin , J. H. Kim , J. H. Ryu , and S. H. Han , “Neurotoxic Effects of Local Anesthetics on Developing Motor Neurons in a Rat Model,” Journal of Clinical Medicine 10, no. 5 (2021): 901. 33668828 10.3390/jcm10050901 PMC7956179

[27] G. Michalettos , F. Clausen , E. Rostami , and N. Marklund , “Post‐Injury Treatment With 7,8‐Dihydroxyflavone Attenuates White Matter Pathology in Aged Mice Following Focal Traumatic Brain Injury,” Neurotherapeutics 22, no. 1 (2025): e00472. 39428261 10.1016/j.neurot.2024.e00472 PMC11742853

[28] H. Yang , L. You , Z. Wang , et al., “Bile Duct Ligation Elevates 5‐HT Levels in Cerebral Cortex of Rats Partly due to Impairment of Brain UGT1A6 Expression and Activity via Ammonia Accumulation,” Redox Biology 69 (2024): 103019. 38163420 10.1016/j.redox.2023.103019 PMC10794929

[29] A. Iyaswamy , A. Thakur , X. J. Guan , et al., “Fe65‐Engineered Neuronal Exosomes Encapsulating Corynoxine‐B Ameliorate Cognition and Pathology of Alzheimer's Disease,” Signal Transduction and Targeted Therapy 8, no. 1 (2023): 404. 37867176 10.1038/s41392-023-01657-4 PMC10590775

[30] H. Liu , X. Wu , J. Luo , et al., “Pterostilbene Attenuates Astrocytic Inflammation and Neuronal Oxidative Injury After Ischemia‐Reperfusion by Inhibiting NF‐κB Phosphorylation,” Frontiers in Immunology 10 (2019): 2408. 31681297 10.3389/fimmu.2019.02408 PMC6811521

[31] T. Wang , Y. Chen , Y. Zou , et al., “Locomotor Hyperactivity in the Early‐Stage Alzheimer's Disease‐Like Pathology of APP/PS1 Mice: Associated With Impaired Polarization of Astrocyte Aquaporin 4,” Aging and Disease 13, no. 5 (2022): 1504–1522. 36186142 10.14336/AD.2022.0219 PMC9466968

[32] P. Goel , S. Chakrabarti , K. Goel , K. Bhutani , T. Chopra , and S. Bali , “Neuronal Cell Death Mechanisms in Alzheimer's Disease: An Insight,” Frontiers in Molecular Neuroscience 15 (2022): 937133. 36090249 10.3389/fnmol.2022.937133 PMC9454331

[33] T. Qiu , Z. Q. Liu , F. Rheault , et al., “Structural White Matter Properties and Cognitive Resilience to Tau Pathology,” Alzheimer's & Dementia 20, no. 5 (2024): 3364–3377. 10.1002/alz.13776 PMC11095478 38561254

[34] Y. C. Kim and K. L. Guan , “mTOR: A Pharmacologic Target for Autophagy Regulation,” Journal of Clinical Investigation 125, no. 1 (2015): 25–32. 25654547 10.1172/JCI73939 PMC4382265

[35] Y. H. Chen , T. Y. Huang , Y. T. Lin , et al., “VPS34 K29/K48 Branched Ubiquitination Governed by UBE3C and TRABID Regulates Autophagy, Proteostasis and Liver Metabolism,” Nature Communications 12, no. 1 (2021): 1322. 10.1038/s41467-021-21715-1 PMC7910580 33637724

[36] Q. Tang , G. Zheng , Z. Feng , et al., “Trehalose Ameliorates Oxidative Stress‐Mediated Mitochondrial Dysfunction and ER Stress via Selective Autophagy Stimulation and Autophagic Flux Restoration in Osteoarthritis Development,” Cell Death & Disease 8, no. 10 (2017): e3081. 28981117 10.1038/cddis.2017.453 PMC5680575

[37] L. Xu , X. Wu , S. Zhao , et al., “Harnessing Nanochaperone‐Mediated Autophagy for Selective Clearance of Pathogenic Tau Protein in Alzheimer's Disease,” Advanced Materials 36 (2024): e2313869. 38688523 10.1002/adma.202313869

[38] E. Chantrapannik , S. Munjupong , N. Limprasert , and S. Jinawong , “Effect of Intravenous Lidocaine on Catheter‐Related Bladder Discomfort, Postoperative Pain and Opioid Requirement in Complex Fusion Lumbar Spinal Surgery: A Randomized, Double Blind, Controlled Trial,” BMC Anesthesiology 24, no. 1 (2024): 405. 39528937 10.1186/s12871-024-02789-y PMC11552165

[39] X. X. Wang , J. Dai , H. W. Deng , Q. Wang , Y. Liu , and H. Guo , “Effect of Intravenous Lidocaine on Postoperative Cognitive Dysfunction in Patients Undergoing General Anesthesia Surgery: A Systematic Review of a Randomized Controlled Trial,” Clinical Therapeutics 47 (2024): 91–101. 39482176 10.1016/j.clinthera.2024.09.027

[40] K. M. Vogt , A. C. Burlew , M. A. Simmons , S. N. Reddy , C. N. Kozdron , and J. W. Ibinson , “Neural Correlates of Systemic Lidocaine Administration in Healthy Adults Measured by Functional MRI: A Single Arm Open Label Study,” British Journal of Anaesthesia 134 (2024): 414–424. 39438214 10.1016/j.bja.2024.07.039 PMC11775839

[41] X. Chen , A. Chen , J. Wei , et al., “Dexmedetomidine Alleviates Cognitive Impairment by Promoting Hippocampal Neurogenesis via BDNF/TrkB/CREB Signaling Pathway in Hypoxic‐Ischemic Neonatal Rats,” CNS Neuroscience & Therapeutics 30, no. 1 (2024): e14486. 37830170 10.1111/cns.14486 PMC10805444

[42] P. Kranke , J. Jokinen , N. L. Pace , et al., “Continuous Intravenous Perioperative Lidocaine Infusion for Postoperative Pain and Recovery,” Cochrane Database of Systematic Reviews 7 (2015): Cd009642. 10.1002/14651858.CD009642.pub2 26184397

[43] H. Kubota , X. Zhang , M. Khalili , X. Zhou , Y. Wen , and T. Nagai , “Automated Behavioral Analysis of Schizophrenia‐Like Phenotypes in Repeated MK‐801‐Treated Mice Using IntelliCage,” International Journal of Molecular Sciences 26, no. 11 (2025): 5184. 40508003 10.3390/ijms26115184 PMC12155132

[44] S. Y. Smith‐Apeldoorn , J. K. E. Veraart , J. Spijker , J. Kamphuis , and R. A. Schoevers , “Maintenance Ketamine Treatment for Depression: A Systematic Review of Efficacy, Safety, and Tolerability,” Lancet Psychiatry 9, no. 11 (2022): 907–921. 36244360 10.1016/S2215-0366(22)00317-0

[45] L. Li , N. Zhu , L. Zhang , et al., “ADHD Pharmacotherapy and Mortality in Individuals With ADHD,” JAMA 331, no. 10 (2024): 850–860. 38470385 10.1001/jama.2024.0851 PMC10936112

[46] C. Niu , X. Yue , J. J. An , R. Bass , H. Xu , and B. Xu , “Genetic Dissection of BDNF and TrkB Expression in Glial Cells,” Biomolecules 14, no. 1 (2024): 91. 38254691 10.3390/biom14010091 PMC10813193

[47] Y. Yang , T. Liu , J. Li , et al., “General Anesthetic Agents Induce Neurotoxicity Through Astrocytes,” Neural Regeneration Research 19, no. 6 (2024): 1299–1307. 37905879 10.4103/1673-5374.385857 PMC11467951

[48] Y. Jia , Y. Song , H. Xue , et al., “Sevoflurane Postconditioning Mitigates Neuronal Hypoxic‐Ischemic Injury via Regulating Reactive Astrocytic STAT3 Protein Modification,” Chemico‐Biological Interactions 405 (2024): 111308. 39536892 10.1016/j.cbi.2024.111308

[49] X. Wu , G. Wen , L. Yan , et al., “Ketamine Administration Causes Cognitive Impairment by Destroying the Circulation Function of the Glymphatic System,” Biomedicine & Pharmacotherapy 175 (2024): 116739. 38759288 10.1016/j.biopha.2024.116739

[50] J. G. Riboldi , J. Correa , M. M. Renfijes , R. Tintorelli , and H. Viola , “Arc and BDNF Mediated Effects of Hippocampal Astrocytic Glutamate Uptake Blockade on Spatial Memory Stages,” Communications Biology 7, no. 1 (2024): 1032. 39174690 10.1038/s42003-024-06586-8 PMC11341830

[51] B. Eroglu , C. Isales , and A. Eroglu , “Age and Duration of Obesity Modulate the Inflammatory Response and Expression of Neuroprotective Factors in Mammalian Female Brain,” Aging Cell 23 (2024): e14313. 39230054 10.1111/acel.14313 PMC11634740

[52] N. M. Mercado , C. Szarowicz , J. A. Stancati , et al., “Advancing Age and the rs6265 BDNF SNP Are Permissive to Graft‐Induced Dyskinesias in Parkinsonian Rats,” NPJ Parkinson's Disease 10, no. 1 (2024): 163. 10.1038/s41531-024-00771-6 PMC11344059 39179609

[53] X. Wang , F. Zhang , L. Niu , et al., “High‐Frequency Repetitive Transcranial Magnetic Stimulation Improves Depressive‐Like Behaviors in CUMS‐Induced Rats by Modulating Astrocyte GLT‐1 to Reduce Glutamate Toxicity,” Journal of Affective Disorders 348 (2024): 265–274. 38159655 10.1016/j.jad.2023.12.068

[54] V. Panwar , A. Singh , M. Bhatt , et al., “Multifaceted Role of mTOR (Mammalian Target of Rapamycin) Signaling Pathway in Human Health and Disease,” Signal Transduction and Targeted Therapy 8, no. 1 (2023): 375. 37779156 10.1038/s41392-023-01608-z PMC10543444

[55] B. Shen , H. Feng , J. Cheng , et al., “Geniposide Alleviates Non‐Alcohol Fatty Liver Disease via Regulating Nrf2/AMPK/mTOR Signalling Pathways,” Journal of Cellular and Molecular Medicine 24, no. 9 (2020): 5097–5108. 32293113 10.1111/jcmm.15139 PMC7205797

[56] P. L. Xie , M. Y. Zheng , R. Han , W. X. Chen , and J. H. Mao , “Pharmacological mTOR Inhibitors in Ameliorating Alzheimer's Disease: Current Review and Perspectives,” Frontiers in Pharmacology 15 (2024): 1366061. 38873415 10.3389/fphar.2024.1366061 PMC11169825

[57] L. He , X. Duan , S. Li , R. Zhang , X. Dai , and M. Lu , “Unveiling the Role of Astrocytes in Postoperative Cognitive Dysfunction,” Ageing Research Reviews 95 (2024): 102223. 38325753 10.1016/j.arr.2024.102223

[58] Y. Chen , G. Wei , X. Feng , E. Lei , and L. Zhang , “Dexmedetomidine Enhances Mitophagy via PINK1 to Alleviate Hippocampal Neuronal Pyroptosis and Improve Postoperative Cognitive Dysfunction in Elderly Rat,” Experimental Neurology 379 (2024): 114842. 38823674 10.1016/j.expneurol.2024.114842

[59] Y. Xiong , W. Liang , X. Wang , et al., “S100A8 Knockdown Activates the PI3K/AKT Signaling Pathway to Inhibit Microglial Autophagy and Improve Cognitive Impairment Mediated by Chronic Sleep Deprivation,” International Immunopharmacology 143, no. Pt 2 (2024): 113375. 39418730 10.1016/j.intimp.2024.113375

[60] L. Feng , H. Lo , J. Zheng , W. Weng , Y. Sun , and X. Pan , “Cycloastragenol Reduces Microglial NLRP3 Inflammasome Activation in Parkinson's Disease Models by Promoting Autophagy and Reducing Scrib‐Driven ROS,” Phytomedicine 135 (2024): 156210. 39522252 10.1016/j.phymed.2024.156210

[61] W. Wang , C. Chen , Q. Wang , et al., “Electroacupuncture Pretreatment Preserves Telomerase Reverse Transcriptase Function and Alleviates Postoperative Cognitive Dysfunction by Suppressing Oxidative Stress and Neuroinflammation in Aged Mice,” CNS Neuroscience & Therapeutics 30, no. 2 (2024): e14373. 37501354 10.1111/cns.14373 PMC10848091

[62] C. Yang , J. Chen , J. Tang , et al., “Study on the Mechanism of Dictyophora Duplicata Polysaccharide in Reducing Depression‐Like Behavior in Mice,” Nutrients 16, no. 21 (2024): 3785. 39519618 10.3390/nu16213785 PMC11547661

[63] S. K. B. Taylor , J. H. Hartman , and B. P. Gupta , “The Neurotrophic Factor MANF Regulates Autophagy and Lysosome Function to Promote Proteostasis in *Caenorhabditis elegans* ,” Proceedings of the National Academy of Sciences of the United States of America 121, no. 43 (2024): e2403906121. 39418305 10.1073/pnas.2403906121 PMC11513987

[64] E. Janda , M. Parafati , C. Martino , et al., “Autophagy and Neuroprotection in Astrocytes Exposed to 6‐Hydroxydopamine Is Negatively Regulated by NQO2: Relevance to Parkinson's Disease,” Scientific Reports 13, no. 1 (2023): 21624. 38062122 10.1038/s41598-023-44666-7 PMC10703796

[65] Y. C. Yoo , N. Y. Kim , S. Shin , et al., “Anti‐Proliferative Effects of Lidocaine as an Autophagy Inducer in Bladder Cancer via Intravesical Instillation: In Vitro and Xenograft Mouse Model Experiments,” Cancers (Basel) 16, no. 7 (2024): 1267. 38610945 10.3390/cancers16071267 PMC11010986

