# Supplementary figures and images for: Repeated Exposure to Lidocaine Induces Alzheimer's‐Like Cognitive Impairment and Neuropathology in Aged Mice Through BDNF‐Regulated Autophagy
Source: J Cell Mol Med. 2025 Nov 29;29(23):e70970. doi: 10.1111/jcmm.70970 (PMC12664910; doi:10.1111/jcmm.70970)

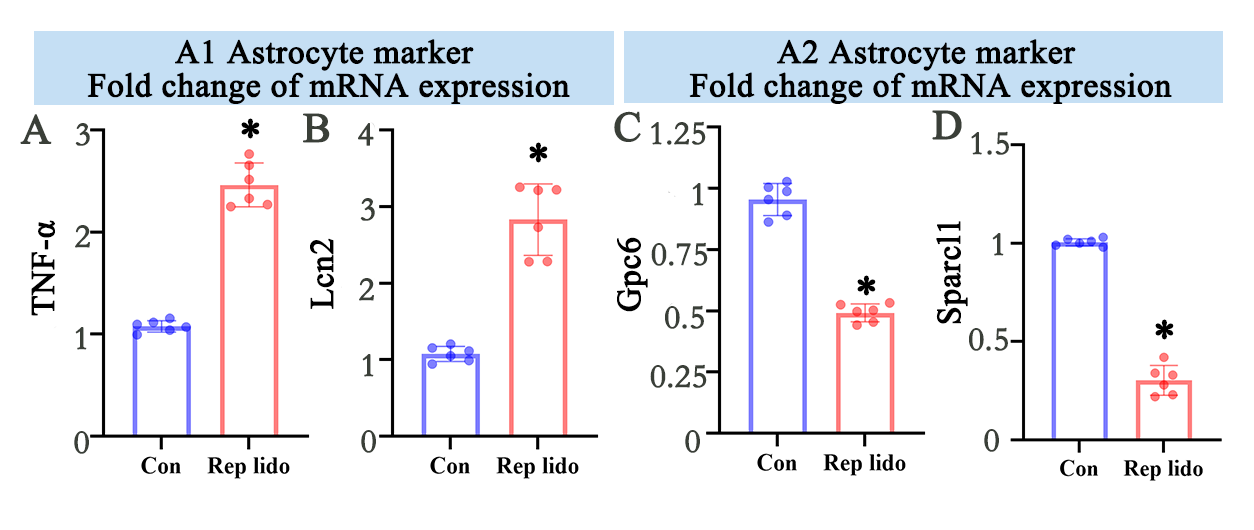

Supplement: Supplementary file 1 — Figure S1: RT‐qPCR analysis of A1 and A2 astrocyte marker expression following repeated lidocaine exposure (A, B) Relative mRNA levels of markers specific to A1 (TNF‐α, Lcn2). (C, D) Relative mRNA levels of markers specific to A2 (Gpc6, Sparcl1). Data were expressed as the mean ± SD (n = 6 per group); *p < 0.05 vs. the Con group. [file JCMM-29-e70970-s002.tif]

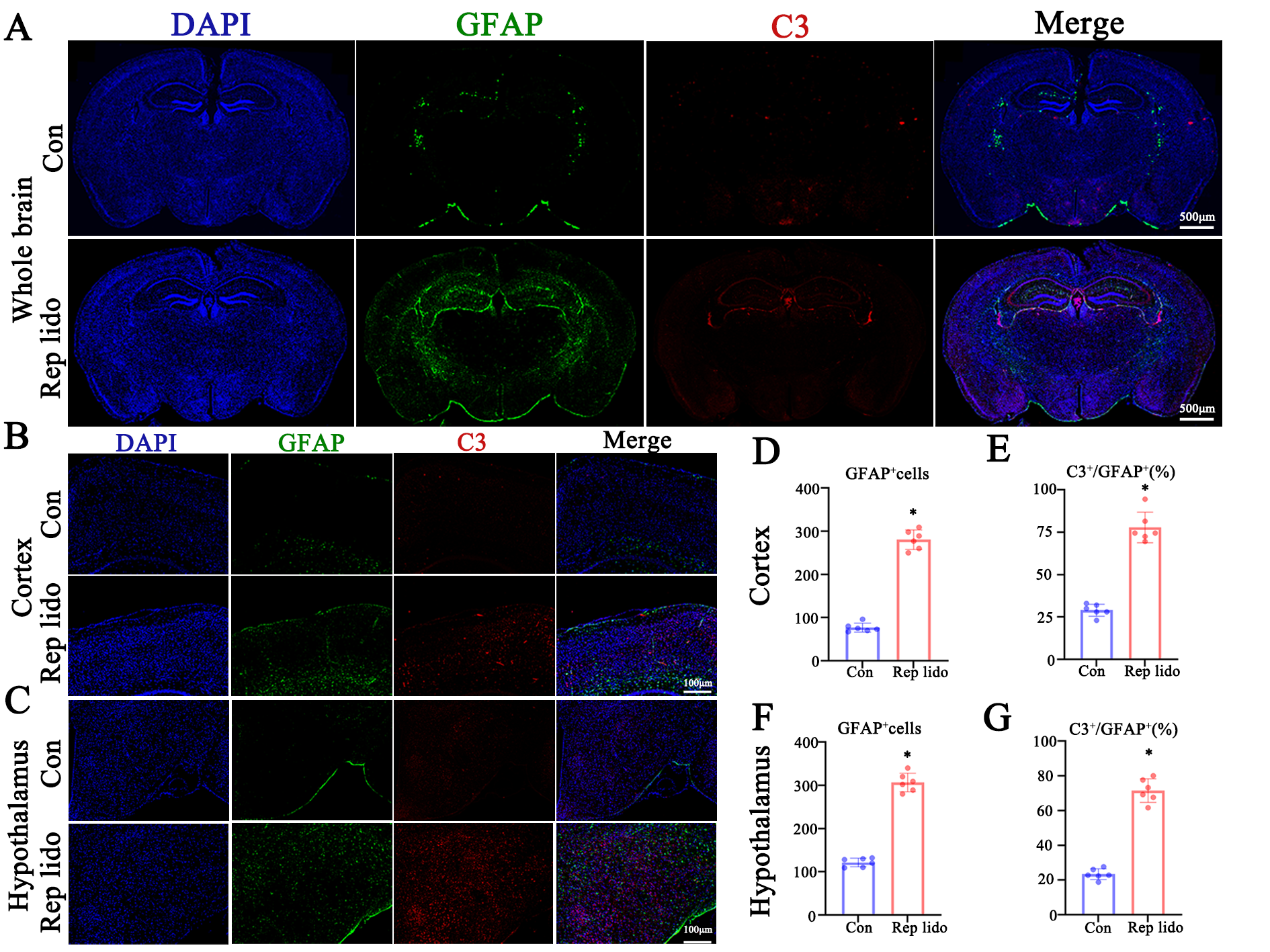

Supplement: Supplementary file 2 — Figure S2: Effects of repeated lidocaine exposure on astrocyte activation in multiple brain regions (A–C) Representative images of GFAP and C3 staining in whole brain, cortex and hypothalamus. (D, E) Quantification of the number of GFAP and C3 positive cells in the Cortex region. (F, G) Quantification of the number of GFAP and C3 positive cells in the hypothalamus region. Data were expressed as the mean ± SD (n = 6 per group); *p < 0.05 vs. the Con group. [file JCMM-29-e70970-s001.tif]
